# Identification of candidate MYB transcription factors that influence *CslF6* expression in barley grain

**DOI:** 10.3389/fpls.2022.883139

**Published:** 2022-09-08

**Authors:** Guillermo Garcia-Gimenez, Miriam Schreiber, George Dimitroff, Alan Little, Rohan Singh, Geoffrey B. Fincher, Rachel A. Burton, Robbie Waugh, Matthew R. Tucker, Kelly Houston

**Affiliations:** ^1^The James Hutton Institute, Dundee, United Kingdom; ^2^Plant Sciences Division, College of Life Sciences, University of Dundee, Dundee, United Kingdom; ^3^School of Agriculture, Food and Wine, The University of Adelaide, Adelaide, SA, Australia

**Keywords:** cell wall, (1,3;1,4)-β-glucan, barley, transcription factors, MYB

## Abstract

(1,3;1,4)-β-Glucan is a non-cellulosic polysaccharide required for correct barley grain fill and plant development, with industrial relevance in the brewing and the functional food sector. Barley grains contain higher levels of (1,3;1,4)-β-glucan compared to other small grain cereals and this influences their end use, having undesirable effects on brewing and distilling and beneficial effects linked to human health. *HvCslF6* is the main gene contributing to (1,3;1,4)-β-glucan biosynthesis in the grain. Here, the transcriptional regulation of *HvCslF6* was investigated using an *in-silico* analysis of transcription factor binding sites (TFBS) in its putative promoter, and functional characterization in a barley protoplast transient expression system. Based on TFBS predictions, TF classes AP2/ERF, MYB, and basic helix-loop-helix (bHLH) were over-represented within a 1,000 bp proximal *HvCslF6* promoter region. Dual luciferase assays based on multiple *HvCslF6* deletion constructs revealed the promoter fragment driving *HvCslF6* expression. Highest *HvCslF6* promoter activity was narrowed down to a 51 bp region located −331 bp to −382 bp upstream of the start codon. We combined this with TFBS predictions to identify two MYB TFs: *HvMYB61* and *HvMYB46/83* as putative activators of *HvCslF6* expression. Gene network analyses assigned *HvMYB61* to the same co-expression module as *HvCslF6* and other primary cellulose synthases (*HvCesA1*, *HvCesA2*, and *HvCesA6*), whereas *HvMYB46/83* was assigned to a different module. Based on RNA-seq expression during grain development, *HvMYB61* was cloned and tested in the protoplast system. The transient over-expression of *HvMYB61* in barley protoplasts suggested a positive regulatory effect on *HvCslF6* expression.

## Introduction

The primary cell walls of certain members of the Poaceae, including barley, are enriched with (1,3;1,4)-β-glucan, a distinctive non-cellulosic polysaccharide that predominantly accumulates during cell expansion (Carpita et al., 2001). In contrast, secondary walls of the Poaceae are mainly characterized by the presence of heteroxylans as the major non-cellulosic component, and tissue-specific lignin accumulation (Burton and Fincher, 2014). This differs from eudicots where xyloglucan and pectins are the most abundant non-cellulosic polysaccharides found in primary cell walls and secondary walls are characterized by the presence of heteroxylans, predominantly 4-O-methylglucuronoxylans, heteromannans and lignin (Harris, 2006).

The discovery of *Cellulose synthase-like* (*Csl*) genes required for (1,3;1,4)-β-glucan biosynthesis, including members of the *CslF*, *H* and *J* families (Burton et al., 2006; Doblin et al., 2009; Little et al., 2018), provided the basis for detailed biochemical and molecular studies of (1,3;1,4)-β-glucan structure, assembly and function. These genetic discoveries complemented initial biochemical and physicochemical studies of (1,3;1,4)-β-glucan carried out predominantly in barley (Woodward et al., 1983), wheat (Bacic and Stone, 1980), and oats (Aaman and Graham, 1987), which reported remarkable differences in relative polysaccharide abundance and structure across cereal species (Bacic and Stone, 1981; Burton and Fincher, 2012). Previous studies identified polymorphisms in the *HvCslF6* gene, a key gene involved in grain (1,3;1,4)-β-glucan biosynthesis (Cory et al., 2012; Taketa et al., 2012; Hu et al., 2014), variations in *CslF* transcript abundance (Burton et al., 2008; Wong et al., 2015), *HvCslF6* subcellular location (Wilson et al., 2015) and amino acids contributing to *HvCslF6* protein structure and (1,3;1,4)-β-glucan solubility (Jobling, 2015; Dimitroff et al., 2016). Despite this, no clear relationship has been established between barley cultivar-specific polymorphisms in the *HvCslF6* upstream region, or the introns of this gene, and the wide range in grain (1,3;1,4)-β-glucan levels observed in barley (Houston et al., 2014; Garcia-Gimenez et al., 2019).

Little is known about regulatory mechanisms affecting primary cell wall formation in grasses, and specifically the biosynthesis of (1,3;1,4)-β-glucan in developing barley grain. In a co-expression analysis of transcribed genes during the cellularization of developing barley endosperm, Zhang et al. (2016a) identified several classes of candidate TF genes that might be involved in cell wall synthesis, but at that stage direct interactions between TF and specific cell wall genes were not investigated. In the model grass *Brachypodium distachyon* a trihelix transcription factor (THX1) was validated as a positive regulator of *BdCslF6* binding to a GT-rich cis-element in the second intron of this gene (Fan et al., 2018). In rice, Zhao et al. (2019) carried out a comprehensive study of transcriptional regulators within the context of cell wall composition. They identified several genes, including members of the MYB gene family, that changed the transcript levels of known cell wall synthesis genes in a transient expression system. One gene, *OsMYB61a*, was of interest due to its ability within this system to regulate the expression of numerous cell wall genes and the observation that knock out lines had altered cell wall composition (Zhao et al., 2019).

Regulation of cell wall composition has also been studied in *Arabidopsis thaliana*, particularly in the context of secondary cell walls. This led to the identification of key transcription factors (i.e., secondary wall NAC; SWN) (Taylor-Teeples et al., 2015) that act as regulatory switches enhancing the expression of downstream MYB master regulators, MYB46 and MYB83 (McCarthy et al., 2009). These MYBs interact with a wide range of cell wall-associated transcription factors (Kim et al., 2014) and networks affecting polysaccharide biosynthesis. In rice and maize, functional orthologs of these *Arabidopsis* secondary cell wall MYB master regulators were identified, suggesting a conserved regulatory mechanism across vascular plants (Zhong et al., 2011; Rao and Dixon, 2018).

Here, our work aimed to explore the transcriptional regulation of *HvCslF6* by identifying putative cis-elements in the 5′ promoter region of *HvCslF6* and the transcription factor(s) binding to them. Such data would provide another avenue to investigate the regulation of *HvCslF6* gene expression and how this affects (1,3;1,4)-β-glucan abundance.

First, the ability of a 3,000 bp *HvCslF6* upstream region to drive expression was tested by GFP tagging in transgenic barley lines. To identify regulatory motifs in the *HvCslF6* promoter, a series of 5′ deletion constructs generated from the 3,000 bp *HvCslF6* upstream region were fused to the dual luciferase reporter system. A barley protoplast-based transient expression system was adapted (Rao, 2007; Yoo et al., 2007) and used to test activity of the *HvCslF6* promoter deletion constructs, permitting the analysis of six deletion constructs simultaneously. Our results from the *HvCslF6* promoter characterization were combined with *in silico* predictions for transcription factor binding sites (TFBS), gene network and co-expression analyses, and previous findings from other cereal species, which suggested several candidate transcription factors that may regulate *HvCslF6* expression.

## Results

### A 3,000 bp *HvCslF6* putative promoter region is sufficient to drive expression in grain and vegetative tissues

**FIGURE 1:**
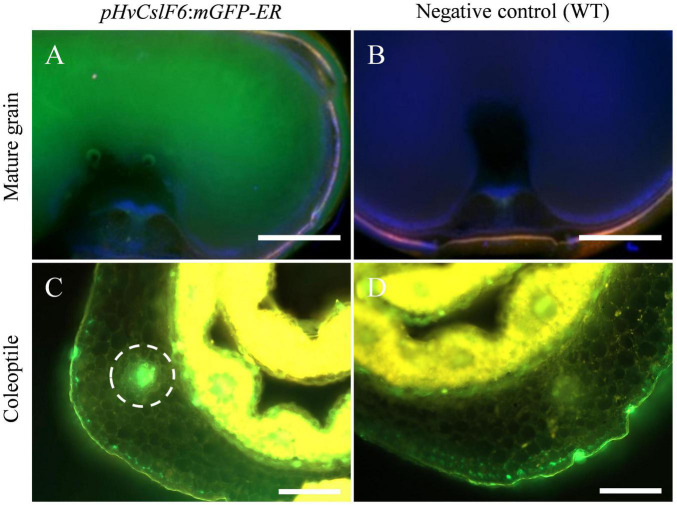
Fluorescent images of *pHvCslF6:mGFP-ER* (–3,000 bp) barley transverse sections in mature grain and coleoptile tissues (cv. Golden Promise). **(A)** Mature grain from line transformed with *pHvCslF6:mGFP-ER*, **(B)** negative control of wild type grain section, **(C)** coleoptile from line transformed with *pHvCslF6:mGFP-ER*, **(D)** negative control of wild type coleoptile section. The positive control is mGFP-ER driven by the 35S CaMV promoter. The dashed circle indicates the location of a vascular bundle. Images were collected on a Zeiss M2 AxioImager equipped with DIC optics and an Apotome.2 (Zeiss, Germany). GFP was excited at 488 nm and emission collected from 499 to 530 nm, in green. Scale bars are 1 mm.

*HvCslF6* is expressed across a range of tissues including grain and young/developing tissues such as the coleoptile, root and leaves (Burton et al., 2008). To study the transcriptional regulation of this gene *in planta*, transgenic barley lines carrying endoplasmic reticulum-targeted GFP (mGFP-ER) driven by a 3,000 bp 5′ sequence upstream of *HvCslF6* (*pHvCslF6*) were generated and screened in grain and vegetative tissues. Transverse sections of transgenic mature grain showed strong mGFP-ER expression in the starchy endosperm (Figure 1A) compared to the negative control (empty vector) (Figure 1B). Similarly, increased mGFP-ER expression around vascular bundles was detected in transverse coleoptile sections of transgenic lines (Figure 1C) compared to the negative control (Figure 1D). Therefore, we confirmed that the 3,000 bp 5′ sequence upstream of *HvCslF6* is sufficient to drive expression of this GFP gene in both grain and vegetative tissues.

### Analysis of transcription factor binding sites in the *HvCslF6* putative promoter region reveals overrepresentation of MYB/SANT, AP2/ERF, and basic helix-loop-helix-related motifs

As the 3,000 bp 5′ promotor region was sufficient to drive the temporal and spatial expression of *HvCslF6* in developing and young tissues, we explored whether known transcription factor binding motifs were either present or enriched within this sequence. First, three TFBS prediction software packages (TRANSFAC^®^ v2014, JASPAR v2020 and PlantPAN v3.0) were compared to identify which produced the most detailed motif predictions with the least number of redundant results. TFBS prediction outputs were compared across the three programs, revealing differences in frequency (number of times a particular motif was predicted within the 3,000 bp *pHvCslF6*) and annotation (profile summary of each motif comprising TF class and origin species) of predicted TFBSs (Supplementary Figure 1 and Supplementary Table 1). It should be noted that a high rate of false positives in predicted TFBSs has been reported in most of these prediction tools (Shahmuradov et al., 2005). Therefore, choosing a comprehensive and experimentally validated database of TFBSs was key to obtain reliable *in silico* predictions. TRANSFAC^®^ was discarded due to the relatively low number of predicted motifs that were also identified using JASPAR and PlantPAN. A comparative motif analysis between PlantPAN and JASPAR identified seven classes of TFBSs with similar relative frequencies (allowing a ± 10% variation in motif prediction results across both programs). These were: MYB/SANT (12.5% in JASPAR and 14.0% in PlantPAN, respectively), DNA-binding with one zinc finger DOF (13.3% and 8.9%), APETALA2/Ethylene response factors AP2/ERF (9.3% and 8.4%), basic leucine zipper bZIP (4.7% and 6.5%), WRKY (5.4% and 3.2%), auxin response factors ARF (B3 domain; 3.1% and 5.2%), and NAC (4.6% and 3.6%). Notably, 52% of PlantPAN predicted motifs could not be classified because no information/description was available regarding their hit sequence.

Based on these results, JASPAR was chosen for subsequent *in silico* analyses of the proximal promoter region of *HvCslF6* (1,000 bp upstream of the start codon) and 355 non-redundant motifs were identified. JASPAR TFBS scores are based on position frequency matrices (PFMs), defined as occurrences of each nucleotide at each position in a set of observed TF-DNA interactions (Fornes et al., 2020). We selected JASPAR for further analyses due to the higher number of non-redundant predicted TFBS and motif-associated information compared to TRANSFAC and PlantPAN. In addition, JASPAR is a regularly maintained open-access, manually curated and experimentally defined database for plant-specific TFBSs (Castro-Mondragon et al., 2022).

**FIGURE 2:**
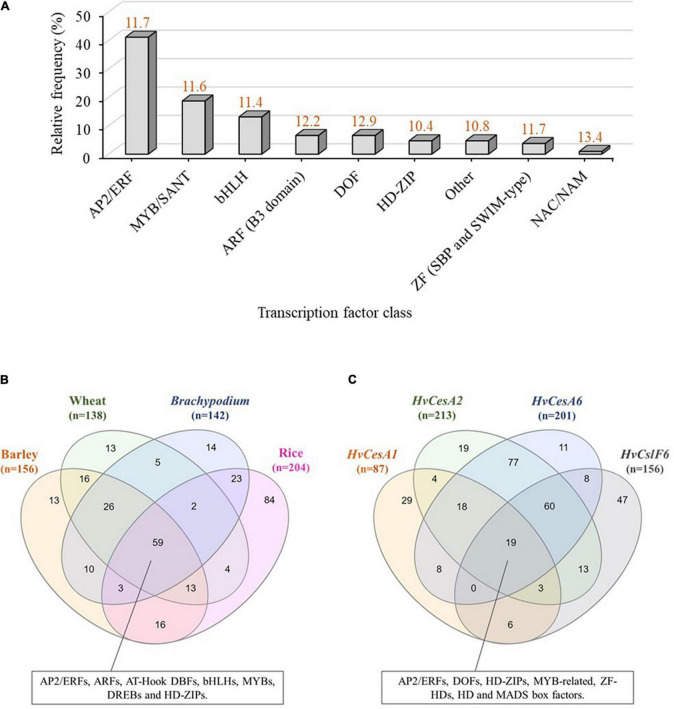
*In-silico* analysis of transcription factor binding sites (TFBSs) in the *CslF6* promoter. **(A)** TFBS predictions identified within a 1,000 bp upstream region of *HvCslF6* start codon using JASPAR **(Fornes et al., 2020)**. TFBS predictions were grouped into TF classes and expressed as relative frequency % of TFBSs corresponding to a particular TF family from the total TFBSs predicted (*n* = 107, after applying *a* ≥ 10 matrix score cut-off to maximize TFBS prediction accuracy). Other TF class indicate a mixed class of low-represented TFBSs (frequency within *HvCslF6* full length promoter ≤ 2%). Average prediction scores for each TF class are shown in orange on top of each bar and were inferred from position frequency matrices (PFMs) and TF flexible models (TFFMs) in JASPAR. **(B)** Venn diagram of TFBS predicted within putative *CslF6* promoter regions (**–**1,000 bp) across barley (*Hordeum vulgare*), wheat (*Triticum aestivum*), *Brachypodium distachyon* and rice (*Oryza sativa*). **(C)** Venn diagram of TFBSs predicted within *HvCesA1*, *HvCesA2*, *HvCesA6*, and *HvCslF6* putative promoter regions (**–**1,000 bp) with identical filtering as described above. Venn diagrams were created using InteractiVenn **(Heberle et al., 2015)**.

These 355 predicted motifs were filtered in accordance with an arbitrary prediction score threshold of ≥10 (maximum score was 15.9) to minimize the detection of false positives/redundant motifs in identical promoter locations (unfiltered TFBS predictions are shown in Supplementary Figure 2). By applying this filter, 107 unique predicted motifs with prediction scores between 0.7 and 15.9 were identified and grouped into eight classes of TFBSs including: MYB/SANT, AP2/ERF, basic helix-loop-helix (bHLH), ARF, DOF, Homeodomain-leucine zipper (HD-ZIP), Zinc Finger (ZF; SBP and SWIM type), NAC/NAM and a mixed class of TFBSs (low-represented putative TF classes with high prediction scores) (Figure 2A). Predicted motifs grouped into AP2/ERF, MYB/SANT and bHLH TF classes were the most abundant, hence over-represented within the *HvCslF6* proximal promoter region (−1,000 bp) when compared to the total of 107 non-redundant motifs identified. JASPAR prediction scores for the three most abundant TF classes were similar to each other (11.6, MYB/SANT; 11.4, bHLH and 11.7, AP2/ERF; Shown on top of each column in orange, Figure 2A).

### Comparative analysis of *CslF6* transcription factor binding sites in other cereal species and in genes co-expressed with *HvCslF6* identifies common motifs for certain transcription factor families

To understand if there was conservation of binding site motifs across grass species, we carried out *in-silico* analysis of the putative *CslF6* promoter sequences in wheat (*Triticum aestivum*), *Brachypodium distachyon* and rice (*Oryza sativa*), and compared the TFBS to those identified in barley (filtered by *a* ≥ 10 prediction accuracy score cut-off, as previously applied to *HvCslF6* full length promoter analysis). This revealed 59 common motifs across all species (Figure 2B and Supplementary Table 2) corresponding to: MYBs/SANTs, ARFs, AP2/ERFs, AT-Hook DNA-binding factors, bHLHs, DREBs, and HD-ZIPs TF classes. The second largest group of common motifs (26) was shared between barley, wheat and *Brachypodium CslF6* upstream sequences and corresponded to putative binding sites for MYB-related TFs, (predominantly MYBs/SANTs: MYB55/61, MYB59, MYB111 and MYB113; GARP/G2-LIKE: PHL11, PHL12; MYB-related: UIF1), and other less abundant bHLH and DOF-related motifs. The rice *CslF6* upstream sequence contained the highest number of unique TFBS predictions (84) compared to other cereals (13, barley; 13, wheat; and 14, *Brachypodium*). The frequency, location, and description of predicted TFBS across all species is shown in Supplementary Table 2.

Next, the analysis of TFBSs was expanded to include three more members of the *Cellulose synthase* gene superfamily, namely *HvCesA1*, *HvCesA2* and *HvCesA6*, which are involved in cellulose biosynthesis in the primary cell wall (Burn et al., 2002; Burton et al., 2006) and known to be co-expressed with *HvCslF6* (Burton et al., 2004; Burton and Fincher, 2009). Common predicted motifs across *HvCesA1*, *HvCesA2*, *HvCesA6*, and *HvCslF6* corresponded to MYB-related, AP2/ERF, HD-ZIP, DOF, ZF-HD, Homeo Domain (HD), and MADS box factors. The largest set of common motifs was found across *HvCesA2* and *HvCesA6* putative promoters (77), followed by *HvCesA2 HvCesA6*, and *HvCslF6* (60) (Figure 2C). As a comparison three *HvCesA* genes (*HvCesA4*, *HvCesA7*, and *HvCesA8*) which are not co-expressed with *HvCslF6* were also analyzed for TFBSs. This analysis identified one common predicted TFBS, a SQUAMOSA promoter-binding protein-like (SPL) motif across all putative promoters (SPL TFs are mainly involved in plant growth and development; Tripathi et al., 2018) and abundant gene-specific, not shared, motif subsets. The largest set of TFBSs was found in the *HvCslF6* promoter (57) containing MYBs, AP2/ERFs HD-ZIP among other TF classes. (Supplementary Figure 3 and Supplementary Table 3).

### Identification of the essential *HvCslF6* promoter region for transcription

**FIGURE 3:**
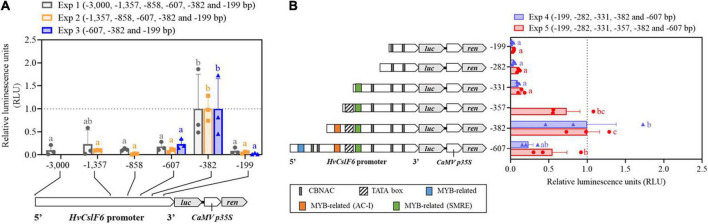
Characterization of the *HvCslF6* promoter using dual luciferase assays to identify region(s) response for increase in expression. Promoter activity in panels **(A,B)** is expressed as relative luciferase activity (luciferase/renilla ratio) and normalized to the –382 construct (with highest promoter activity, dashed gray line) on each experiment, respectively. Three independent protoplasts transfections (biological replicates) were performed per deletion construct and averaged to calculate mean reporter activity 24 h after transfection for each experiment. Negative controls (*n* = 3 empty vector lacking *HvCslF6* promoter) were performed in parallel and luciferase background activity subtracted from tested constructs (*n* = 3 dual luciferase assays per construct, as technical replicates). **(A)** *HvCslF6* promoter activity analyzed in six deletion constructs (from –3,000 bp full length promoter to –199 bp) using dual luciferase assays across three independent experiments (Exp1, Exp2, and Exp3). Letters above each construct indicate significant differences within each experiment determined by one-way ANOVA (*p*-value 0.041, Exp1; *p*-value < 0.01, Exp2 and *p*-value 0.027, Exp3) followed by Tukey’s multi-comparison test. Error bars represent standard error (*n* = 3). **(B)** *HvCslF6* promoter activity of 5′ *HvCslF6* promoter deletion constructs from –607 bp to –199 bp in barley protoplasts. Gray bar indicates CBNAC calmodulin-binding NAC motif, blue bars indicate MYB-related motifs, yellow bars denote MYB-related AC-I motifs, green bars indicate secondary wall MYB-responsive element, (SMRE) and the diagonally stripped bar denotes predicted location of the TATA box consensus sequence. Different letters above each construct (i.e., a,b) indicate significant differences determined by one-way ANOVA (*p*-value < 0.01) followed by Tukey’s test. Error bars represent standard error (*n* = 3).

To identify the *HvCslF6* promoter region necessary for transcriptional activation, seven progressively deleted promoter constructs were generated from the 3,000 bp region upstream of the *HvCslF6* start codon (−3,000, −1,846, −1,357, −858, −607, −382, and −199 bp) and tested in a barley leaf-derived protoplast system (previously optimized using GFP/YFP-expressing constructs; Supplementary Figure 4). The region of the promoter that produced the highest relative luciferase assay activity was a −382 bp region upstream of the *HvCslF6* start codon (Figure 3A). Additionally, deletion constructs containing a −607 bp or −199 bp *HvCslF6* promoter region showed a significant decrease in promoter activity based on the relative luciferase assay compared to the −382 bp region. Therefore, we had delimited a 183 bp sequence within the proximal *HvCslF6* promoter between the two smallest deletion constructs (from −382 to −199 bp) that showed a relative increase in luciferase activity and could be responsible for upregulation of *HvCslF6* expression. *HvCslF6* promoter activity was comparatively low from −607 to −3,000 bp upstream of the *HvCslF6* CDS, indicating that repressors of *HvCslF6* expression could be binding to this −382 to −607 bp region. Furthermore, no significant differences in promoter activity were observed across constructs carrying the longest *HvCslF6* promoter fragments (−3,000, −1,846, and −1,357 bp; Supplementary Figure 5).

We retrieved *HvCslF6* proximal promoter regions (1,000 bp upstream from start codon) from cv. Morex and cv. Golden Promise and carried out a pairwise sequence alignment. Both promoters were identical based on the barley pangenome sequences available at GrainGenes^1^ therefore subsequent *in silico* prediction of TF binding sites could also be extended to cv. Golden Promise.

To further delimit putative TF binding sites within the region that showed increased luciferase activity, three additional deletion constructs were generated. Constructs containing −282 bp, −331 bp, and −357 bp of the *HvCslF6* promoter, were tested alongside three previously tested deletion constructs (−199 bp, −382 bp, and −607 bp) to normalize dual luciferase measurements and compare promoter activity across deletion constructs (Figure 3B). A significant increase in promoter activity (*p*-Value 0.029, Tukey′s test) was detected in the −357 bp deletion construct. *In-silico* prediction of this promoter fragment indicates that it includes a putative TATA box element (5′–TATAAA–3′). This likely explains the increase in *HvCslF6* promoter activity with the inclusion of this region of the promoter, and the low/zero levels of promoter activity in the first three constructs which based on our *in-silico* prediction lack a TATA box. Highest *HvCslF6* promoter activity was narrowed down to a 51 bp region located −331 bp to −382 bp upstream of the start codon, although no significant differences (*p*-Value 0.321, Tukey’s test) were determined between the −357 bp and −382 bp deletion constructs. Moreover, PlantPAN and JASPAR *in-silico* motif analysis of the additional 25 bp included in the −382 bp fragment compared to the −357 bp fragment predicted a single MYB-related motif, AC-I, enriched in AC bases. Results also indicated low *HvCslF6* promoter activity from −199 bp to −331 bp based on three deletion constructs analyzed (−199 bp, −282 bp, and −331 bp). This region contained two (three in the case of −331 bp construct) predicted calmodulin-binding NAC elements and a MYB-related motif, described as a secondary wall MYB-responsive element (SMRE) based on JASPAR analysis (Figure 3B).

### MYB transcription factor binding sites are located within a promoter region showing increased luciferase activity

Based on previous studies which suggest a key role for NAC and MYB TF families in SCW polysaccharide biosynthesis (Taylor-Teeples et al., 2015) and the involvement of MYB TFs in grass cell wall synthesis including hemicelluloses (Zhao et al., 2019), we mapped two putative MYB-related *cis*-elements (among other predicted motifs from our *in-silico* analysis) onto *HvCslF6* promoter regions that conveyed increased promoter activity (Figure 3B and Table 1). A six-nucleotide, AC-I element (5′–ACCTAC–3′) was predicted from −364 to −358 bp upstream from the *HvCslF6* start codon. Prouse and Campbell (2013), demonstrated that the R2R3-MYB *AtMYB61* can bind to this AC-I element, therefore we were interested in the barley ortholog of this gene as a potential regulator of *HvCslF6* expression. A putative binding site in construct −331 (5′–CGTTGGT–3′/3′–ACCAACG–5′) corresponds to another MYB consensus motif, also described as a secondary wall MYB-responsive element (SMRE) or a MYB46-responsive cis-regulatory element, M46RE (Zhong and Ye, 2012). This *cis*-element was detected in reverse orientation (3′–5′) and in *Arabidopsis* the same motif can be bound by AtMYB46 and AtMYB83 (Kim et al., 2012; Ko et al., 2014). Additionally, a secondary wall MYB-responsive element (SMRE) motif was predicted from −299 to −292 bp, downstream of the TATA box in the *HvCslF6* promoter (see construct −331, Figure 3B). However, this had limited impact on promoter activity, likely due to the absence of the TATA box.

**TABLE 1:** Description of putative binding sites found in the *HvCslF6* promoter based on dual luciferase results of deletion constructs with differential promoter activity.

Putative motif/s in p*HvCslF6* (5′–3′)	*Cis*-acting element/s (5′–3′)	Description	Candidate TF	References
ACCTAC (−364 bp)	MYB consensus: ACC(A/T)A(A/C)	AC-I element, bound by R2R3-MYB proteins	HvMYB61	Romano et al., 2012; Prouse and Campbell, 2013; Zhao et al., 2019
CGTTGGT (−299, −235, −364, −386, −478 bp) (3′–ACCAACG–5′)	MYB consensus/SMRE: ACC(A/T)A(A/C) (T/C)	Secondary wall MYB-responsive element (SMRE)	HvMYB46/83	Kim et al., 2012; Zhong and Ye, 2012
	MYB consensus/M46RE: (T/C)ACC(A/T)A(A/C) (T/C)	MYB46-responsive cis-regulatory element (M46RE)		Ko et al., 2014
GGTAGGTAGGT (−478 bp) (5′–GGTAGGT–3′) (3′–ACCTACC–5′)	R2R3-MYB/MYB3: GGTAGGT(A/G) MYB consensus/SMRE: ACC(A/T)A(A/C) (T/C)	MYB3; subgroup S4 AC-I/Secondary wall MYB-responsive element (SMRE)	R2R3-MYB/s	Dubos et al., 2010; Ko et al., 2014

To assess protein sequence similarities in MYB46, MYB83, and MYB61 orthologs between barley (Table 2), *Arabidopsis* (Romano et al., 2012) and rice (Zhong et al., 2011), we constructed an unrooted phylogenetic tree of R2R3-type MYB transcription factors which includes those that could putatively bind to the −382 bp *HvCslF6* promoter region (Supplementary Figure 6). The candidate MYB transcription factors that correspond to the predicted binding sites belong to the plant specific R2R3-MYB subfamily, which is characterized by the presence of two highly conserved MYB DNA-binding domains (Pfam PF00249; Bateman et al., 2017) and constitutes the largest MYB subgroup. HvMYB61 was grouped together with their orthologs from *Arabidopsis* (AtMYB61; Prouse and Campbell, 2013) and rice (OsMYB61a and OsMYB61b; Zhao et al., 2019). The best fitting barley ortholog for AtMYB46 and AtMYB83 was a single gene named HvMYB46/83, following previous studies in rice (OsMYB46/83; Rao and Dixon, 2018; Zhao et al., 2019) and grouped with its orthologs (Supplementary Figure 6).

**TABLE 2:** Description of candidate transcription factors retrieved from GrainGenes (https://wheat.pw.usda.gov/GG3/) based on cv. MorexV3 **(Mascher et al., 2021)**.

Candidate TF	Barley gene ID	Position	5 DPA FPKM	15 DPA FPKM
*HvMYB46/83*	HORVU.MOREX. r3.5HG0447760	chr5H:141063373. .141065705	0.03	0.00
*HvMYB61*	HORVU.MOREX. r3.1HG0018590	chr1H:61436701. .61439073	4.49	0.89

Expression data (fragments per kilobase of exon per million, FPKM across three biological replicates) in developing barley grain at 5 and 15 days post anthesis (DPA), were retrieved from barleyGenes RNA-Seq Database (Available from: https://ics.hutton.ac.uk/barleyGenes/index.html).

### Gene network analysis of candidate transcription factors

Using gene expression data from 808 individual samples (Supplementary Table 4; Milne et al., 2021), we constructed a co-expression network to identify genes closely connected to *HvCslF6*. The samples covered a wide range of biotic and abiotic stresses, tissue types and cultivars, resulting in a robust dataset. Low expressed genes were removed (see section “Materials and methods”) and a weighted gene correlation network analysis (WGCNA) performed (Langfelder and Horvath, 2008). We identified 33 modules, with a color assigned to each module, plus one module (gray) which contains the unallocated genes. *HvCslF6*, along with the primary cell wall cellulose synthases (*HvCesA1*, *HvCesA2*, *HvCesA6*) were assigned to the yellow module (Supplementary Figure 7). *HvMYB61* (one of the candidate *HvCslF6* regulators) and *HvTHX1*, the barley ortholog of *BdTHX1*, which binds to the intronic region of *BdCslF6* and acts as a positive regulator of expression of this gene (Fan et al., 2018), were all members of the yellow module. Visualization of the yellow network in Gephi showed a gene network comprising 992 genes and 54,090 gene connections (Supplementary Figure 8). To better investigate the connections, an intermediate stringency threshold of 0.188 (the correlation ranged from 0.15 to 0.335) was applied to define a subnetwork of the top 50 genes connected to *HvCslF6*.

**FIGURE 4:**
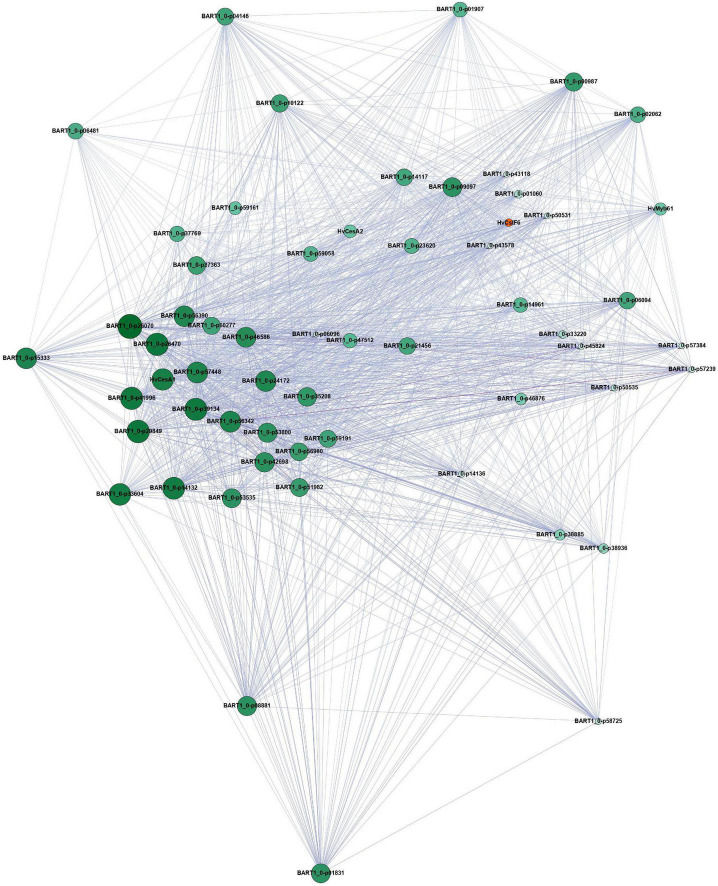
Filtered yellow module network based on genes connected to *HvCslF6* (*n* = 50), visualized using Gephi. This subnetwork was obtained from the publicly available EoRNA database (Milne et al., 2021). Filtering parameters are described in the corresponding methods section. Node size and color correspond to gene degree centrality. Big and dark green nodes have a high number of linked nodes, while small and light green nodes only have a small number of linked nodes. The edges are colored by weight. A dark purple corresponds to a high weight and therefore correlation, while a light purple color corresponds to a lower weight and less strong correlation between the nodes. *HvCslF6* is highlighted in orange. Gene names correspond to the description in Supplementary Table 5. Genes not listed in the table do not contain protein annotation and are potential non-coding genes.

This subnetwork based around *HvCslF6* still included *HvMYB61* (BART1_0-p01380 and the three cellulose synthases; *HvCesA1, HvCesA2, HvCesA6*, BART1_0-p60277, BART1_0-p40943, BART1_0-p44934) (Figure 4). All are either known to be involved in primary cell wall synthesis in barley, or in the case of *HvMYB61* the ortholog of *OsMYB61* has been shown to influence to cell wall synthesis (Zhao et al., 2019). Additionally, this module contained other proteins that have been linked to cell wall processes (Supplementary Table 5). *COBRA-like protein 3* (BART1_0-p39134) located on chromosome 5H was shown to be co-expressed with *HvCesA1*, *HvCesA2*, *and HvCesA6* (Houston et al., 2015). The other candidate regulator, *HvMYB46/83* did not pass the filtering threshold when removing genes with low levels of expression or were assigned to the black instead of yellow module. Hence, this candidate regulator is unlikely to be a global regulator of *HvCslF6*.

**FIGURE 5:**
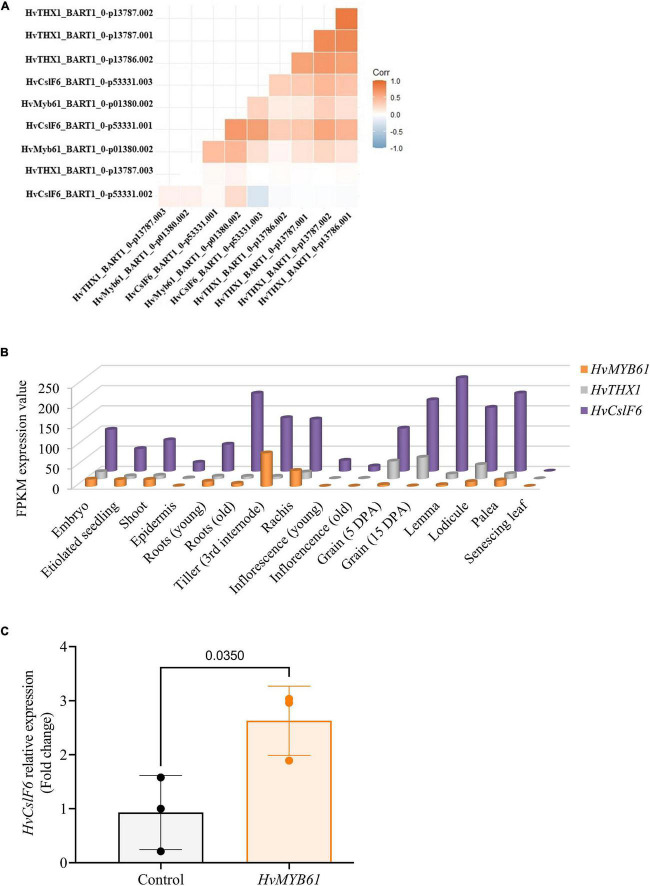
Expression profiles of candidate upregulators of *HvCslF6* expression in several datasets. **(A)** Correlation between TPM values of selected candidate genes from the EoRNA database ordered based on hierarchical clustering. **(B)** Expression levels of *HvMYB61* candidate transcription factor, *HvTHX1* and *HvCslF6* across 16 barley tissues obtained from barleyGenes RNA-seq database. (Available from: https://ics.hutton.ac.uk/barleyGenes/, The James Hutton Institute), expressed as FPKM (fragments per kilobase of exon per million fragments mapped) across three biological replicates per tissue. **(C)** Relative expression of *HvCslF6* in barley protoplasts transfected with an empty vector and over-expressing *HvMYB61*, respectively, after 24 h. Internal control genes: α-*tubulin*, *GAPDH* and *HSP70* were used for normalization of relative expression (2^–ΔΔ*CT*^ method; Vandesompele et al., 2002). Error bars represent standard deviation associated with three independent experiments (transfection assays *n* = 3). qRT-PCR reactions were performed in triplicate. *P*-value was calculated based on Student’s *t*-test (two-tailed).

Then we carried out a correlation analysis using transcript expression data from the eoRNA database (Milne et al., 2021), which contains 22 publicly available datasets and >800 RNA-seq datasets from a wide range of tissues, development stages and experimental treatments. We focused on two subsets of genes. The first included our candidate TF gene *HvMYB61*, a selection of genes from the network analysis and others known to be co-expressed with *HvCslF6* (i.e., *HvCesA1, HvCesA2*, and *HvCesA6*). The second subset was comprised of secondary cell wall genes not co-expressed with *HvCslF6* (i.e., *HvCesA4, HvCesA7, HvCesA8*), and included other members of the *HvCslF/H/J* gene family, and *HvTHX1*, the barley ortholog of *BdTHX1*, a positive regulator of *BdCslF6* (Figure 5A and Supplementary Figure 9). We hypothesized that if *HvMYB61* shows comparable or greater correlation with *HvCslF6* compared to *HvCesA1, HvCesA2, HvCesA6*, this would provide further confidence in a relationship between these candidates. However, if strong positive correlation is observed between the expression of *HvMYB61*, our candidate gene, and the group considered to be secondary cell wall *HvCesA* genes (*HvCesA4, HvCesA7*, and *HvCesA8*), which show no correlation with *HvCslF6*, it might suggest that our candidate gene is unlikely to be involved in the broader regulation *HvCslF6* expression.

For the transcripts of genes considered to be primary cell wall related *CesA* genes and *HvCslF6*, we observed Pearson correlation coefficients of between *r* = 0.9 to −0.1, while the secondary cell wall *CesA* genes had *r* = 0.4 to −0.2 (Supplementary Figure 9). The range of values is not only due to several genes being compared but also multiple transcripts per gene. For several transcripts there was a high degree of correlation between the expression profile for *HvMYB61* and *HvCslF6* (Person correlation coefficient *r* = 0.7 to 0.1) and *HvTHX1* and *HvCslF6* (Person correlation coefficient *r* = 0.6 to −0.1). Therefore, this analysis from an extensive range of samples and conditions provides additional evidence supporting the similar expression dynamics of *HvMYB61* and *HvCslF6*.

To further investigate the expression profile of candidate MYB transcription factors we surveyed several smaller transcript datasets from barley that are independent of those described above. An RNA-seq dataset from cv. Morex across 16 barley tissues showed that *HvMYB61* is expressed abundantly across the entire barley plant (Figure 5B). *HvMYB61* expression was detected in 10-day seedling, shoot (10 cm), tiller (3rd internode), rachis, developing grain at 5 days post anthesis (DPA) and embryo; these are tissues where (1,3;1,4)-β-glucan is present. Similarly, *HvTHX1* is also expressed in multiple tissues including developing grain (5 and 15 DPA). Expanding the RNA-seq expression data across barley grain development, an independent expression dataset based on six different spring barley cultivars (cvs. Hopper, Sloop, Extract, Taphouse, Alabama, and Pewter; Aubert et al., 2018; Matros et al., 2021) was used to retrieve the expression profiles of the candidate genes from early to mid-late grain development. *HvMYB61* was mainly expressed during early grain development, from 7 to 11 DPA, whereas *HvMYB46/83* expression remained almost undetectable from 7 to 20 DPA. In the RNA-seq dataset (developing grain) Pearson correlation coefficients with *HvCslF6* were: 0.95 for *HvMYB61* and 0.98 for *HvTHX1* (showing abundant expression from 7 to 20 DPA; Supplementary Table 6). Our results showed that the positive correlation between *HvMYB61* and *HvCslF6* across different tissues and datasets, in the context of HvTHX1 (ortholog of BdTHX1, known activator of BdCslF6; Fan et al., 2018) and other *HvCesA*s (co-expressed and not co-expressed), are consistent with a regulatory effect of HvMYB61.

### Candidate transcription factor over-expression in barley protoplasts impacts *HvCslF6* expression

Based on expression profiles, our luciferase assay results and data from gene network analyses, we focused subsequent assays on the *HvMYB61* transcription factor, since evidence suggested this gene may influence *HvCslF6* expression. The candidate regulator *HvMYB46/83* was ruled out based on low levels of expression from gene co-expression analyses. The candidate transcription factor *HvMYB61* was cloned and transfected into barley protoplasts plus an empty vector control, respectively. After 24 h, protoplasts were harvested and *HvCslF6* relative transcript abundance was measured. We observed a significant increase in *HvCslF6* expression in the presence of *HvMYB61*, representing a fold change of 2.63 ± 0.64 in *HvCslF6* expression compared to protoplasts transfected with an equal amount of empty vector (*p*-Value 0.035, Student’s *t*-test; Figure 5C). Taken together, our results from the functional characterization of the *HvCslF6* promoter, TF identification and gene co-expression analyses, indicate that the *HvMYB61* transcription factor may be a positive regulator of *HvCslF6* expression in barley.

## Discussion

The transcriptional regulation of primary cell wall formation and in particular (1,3;1,4)-β-glucan biosynthesis, which is present across grasses including many economically important crop species, remains largely unresolved. The interest in the identification of regulators able to fine-tune *HvCslF6* expression and grain (1,3;1,4)-β-glucan content ultimately arises from the beneficial health effects associated with (1,3;1,4)-β-glucan-rich diets (Bai et al., 2019) and its importance for efficient brewing and distilling (Gupta et al., 2010). Several transcription factor families (predominantly NACs and MYBs) are known to regulate polysaccharide biosynthesis in secondary cell walls, some of them with conserved functions between grasses and *Arabidopsis* (McCarthy et al., 2009; Zhou et al., 2009; Ko et al., 2014).

We delimited a region of the functional promoter of *HvCslF6* which contained a binding site motif for *MYB61*. We observed that when this gene was overexpressed in the presence of *HvCslF6* it led to an increase in *HvCslF6* expression relative to control samples which lacked *HvMYB61*. This reflects the results of Zhao et al. (2019) who observed that *OsCslF6* and other cell wall related genes were upregulated in rice protoplasts overexpressing *OsMYB61.* The authors also reported a 31% decrease in leaf (1,3;1,4)-β-glucan content was observed in *OsMYB61a* knockout mutants compared to WT. Barley and rice have distinct patterns of (1,3;1,4)-β-glucan distribution throughout the plant, with barley having relatively high levels of this polysaccharide in the grain compared to vegetative tissue (Burton and Fincher, 2014); the opposite is true of rice with extremely low levels of (1,3;1,4)-β-glucan present in the grain (Vega-Sanchez et al., 2012). Phenotypic analyses of *AtMYB61* loss- and gain-of-function mutants showed similar results to *OsMYB61* RNAi lines (Hirano et al., 2013), confirming a role for this gene in secondary cell wall formation in *Arabidopsis*, in addition to regulating other traits (Penfield et al., 2001; Newman et al., 2004; Romano et al., 2012; Romero-Romero et al., 2018). At present, evidence for a direct interaction between HvMYB61 and the putative binding site in the promoter of *HvCslF6* is lacking, as we did not carry out a yeast-1-hybrid experiment. Although Zhao et al. (2019) did not observe any evidence of direct binding between OsMYB61 and *OsCslF6*, it is important to consider that the levels of (1,3;1,4)-β-glucan are typically 5-fold lower in rice than barley, and hence the expression or function of regulatory genes might be different between these two species.

Unlike rice, which contains two MYB61 genes (OsMYB61a and OsMYB61b; Zhao et al., 2019), *HvMYB61* was only present as a single gene on barley chromosome 1H at 47.8 cM. Notably, this co-located with an association peak for grain (1,3;1,4)-β-glucan content (48.4 cM) which was identified in a previous genome wide association scan using elite barley germplasm (Houston et al., 2014). A QTL for malt (1,3;1,4)-β-glucan was detected in the same genomic region using a cv. Steptoe × Morex population (Han et al., 1995). Other genes described in this region of chromosome 1H are *HvCslF9*, a putative grain (1,3;1,4)-β-glucan synthase based on sequence similarity to other *Csl* genes, and *HvGlbI*, a (1,3;1,4)-β-glucan endohydrolase. *HvGlbI* has been shown to hydrolyse both malt (1,3;1,4)-β-glucan and (1,3;1,4)-β-glucan from germinating grains (Slakeski and Fincher, 1992; Betts et al., 2017) and therefore is a plausible candidate for contributing to variation underlying the QTL’s mentioned above (Han et al., 1995; Houston et al., 2014). However, because knockout mutants for *HvCslF9* exhibit similar (1,3;1,4)-β-glucan content to wild-type barley grain, it seems likely that this gene plays a minor role, if any, in determining mature grain (1,3;1,4)-β-glucan content (Garcia-Gimenez et al., 2020). Hence, while *HvGlbI* and *HvCslF9*’s contribution to grain (1,3;1,4)-β-glucan content is at least already partially understood,*HvMYB61* appears to be an enticing candidate for further investigations of variation in *HvCslF6* expression during grain development.

We also identified another R2R3-MYB binding site located from −299 to −292 bp in the *HvCslF6* promoter. This was functionally characterized as a secondary cell wall MYB-responsive element, SMRE which can be bound by MYB46 and MYB83 in *Arabidopsis thaliana*, regulating secondary wall biosynthesis (Kim et al., 2012; Zhong and Ye, 2012). This SMRE motif was also described as a MYB46-responsive *cis*-regulatory element, M46RE (Ko et al., 2014). However, *HvMYB4/83* mRNA levels were barely detectable in most barley tissues, inconsistent with a major role in controlling (1,3;1,4)-β-glucan content. In contrast, *HvMYB61* expression was detected across several tissues where (1,3;1,4)-β-glucan is present, (and *HvCslF6* is expressed) including early stages of grain development when (1,3;1,4)-β-glucan biosynthesis is occurring. No significant differences in promoter activity were observed for constructs carrying the longer *HvCslF6* promoter fragments (−3,000, −1,846 and −1,357 bp; Supplementary Figure 5), with these fragments generating comparatively low levels of promoter activity. One of these fragments encompassed the section of the *HvCslF6* promoter (from −1,560 to −1,567 bp of the *HvCslF6* start codon) which Wong et al. (2015) had identified an 8 bp insertion in cv. TR251. However, this deletion was absent from all other cultivars in Wong et al. (2015) and the present study.

We observed further support for a relationship between *HvMYB61* and *HvCslF6* from a comprehensive gene network analysis of *HvCslF6* using 807 gene expression sets from different developmental stages and treatments. This analysis assigned *HvMYB61* to the same co-expression module as *HvCslF6*, the primary cellulose synthases (*HvCesA1*, *HvCesA2*, and *HvCes6*), and *HvTHX1*, the ortholog of a known enhancer of *BdCslF6* expression (Fan et al., 2018), consistent with our hypothesis that *HvMYB61* could act as positive regulator of *HvCslF6* expression. Subsequent analysis of *HvMYB61* by transient over-expression in a barley protoplast system led to a significant increase in *HvCslF6* expression in the presence of *HvMYB61* compared to protoplasts transfected with an equal amount of empty vector across three independent experiments. In line with the study from Zhao et al. (2019) in rice, our results suggest that in cereals, *MYB61* may have a conserved role in regulating the expression of grass-specific cell wall biosynthetic enzymes including *HvCslF6*.

The focus of the current study was to delimit putative cis-elements and transcription factors controlling the expression of *HvCslF6*, and to use these findings to further refine our knowledge of what determines (1,3;1,4)-β-glucan content and ultimately provide diagnostics to enable selection for variation in this trait. Based on our findings, we speculate that selected MYB transcription factors may act as positive regulators of (1,3;1,4)-β-glucan accumulation in barley primary cell walls. This relationship relates predominantly to *HvMYB61* and the proximal promoter region of *HvCslF6* and is supported by co-expression in a range of tissues including the young endosperm. However, we anticipate that the regulation of *HvCslF6* expression could be tissue- and genotype-specific. We previously demonstrated that *HvCslF6* expression is genotype-dependent in a relatively genetically narrow selection of barley cultivars (Garcia-Gimenez et al., 2019). Additionally, the temporal and spatial variation of the expression of this gene has been well characterized previously (Burton et al., 2008). Therefore, expanding this work to include a wider set of germplasm, tissues and generating expression profiles for additional genes such as *HvMYB61* would provide additional insight into the relationship between these genes.

The decrease in *HvCslF6* promoter activity observed in the −607 bp construct compared to the −382 bp construct requires further investigation. Recent studies also describe the emerging role of MYBs as repressors, negatively affecting secondary cell wall biosynthesis (Zhao and Bartley, 2014; Rao and Dixon, 2018) and cold acclimation (Zhang et al., 2016b), among other traits (Zhou et al., 2017; Ma and Constabel, 2019). It is also possible that repressive cis-elements are present in the *HvCslF6* promoter. The dual luciferase system allowed us to screen a more distal promoter region from −382 to −607 bp, which showed comparatively low levels of luciferase activity. This suggests that other potential regulatory factors may bind to the *HvCslF6* promoter in the protoplast system, inhibiting the expression of this gene. TFBS predictions in this 225 bp region correspond to several TF families and include additional MYB-related elements. Additionally, it will be intriguing to assess the function of *THX1* barley orthologs, and any potential additive effect with *HvMYB61*.

## Materials and methods

### p*HvCslF6*:mGFP-ER transgenic lines

Barley transgenic lines (cv. Golden Promise) carrying a 3,000 bp upstream region from *HvCslF6* start codon fused to a endoplasmic reticulum-targeted GFP (p*HvCslF6*:mGFP-ER) construct were generated via *Agrobacterium*-mediated transformation at The University of Adelaide following the protocol outlined in Burton et al. (2011). Transgenic lines (T_2_) were screened using a Zeiss Axioskop 2 Plus microscope (Carl Zeiss Microscopy GmbH, Jena, Germany) equipped with an external UV light source (HBO 100). mGFP was excited at 488 nm and emission collected at 500–530 nm. For coleoptile screening, lines were grown in a petri dish with filter paper for 3–4 days in the dark and screened 5 days after gemination. Transverse section images were captured with an AxioCam 512. Subsequent to collection, images were processed with Zen Software v6.0, utilizing global adjustment tools only.

### Transcription factor binding sites prediction in barley and other species

A 3,000 bp region upstream of the *HvCslF6* start codon (cv. Morex) was retrieved from the barley genome explorer (Mascher et al., 2021) and used for TFBS prediction comparison using TRANSFAC^®^ v2014 (Matys et al., 2006), JASPAR v2020 (Fornes et al., 2020) and PlantPAN v3.0 (Chow et al., 2019). Motif over-representation analysis was carried out using *HvCslF6* TFBS predictions from JASPAR and filtered based on ≥ 10.0 prediction score (maximum score = 15) to minimize false positive motif predictions. Upstream *CslF6* sequences from wheat (*Triticum aestivum*), *Brachypodium distachyon* and rice (*Oryza sativa*) were retrieved from Ensembl Plants^2^. TFBS comparisons across species were carried out within a 1,000 bp *CslF6* putative promoter region using JASPAR (Fornes et al., 2020) and predicted motifs filtered based on ≥ 10.0 prediction score. The same filter was also applied for the TFBS analysis of *HvCesA* upstream regions (1,000 bp). Venn diagrams were created using InteractiVenn (Heberle et al., 2015).

### Plant material for protoplast assays

Barley plants (cv. Golden Promise) were grown in a growth chamber with a 16-h light/8-h dark photoperiod at the Functional Genomics (FUNGEN) facility, at The James Hutton Institute, United Kingdom. After 3 weeks, 0.25 g of primary leaves were harvested for protoplast assays. Leaves were cut lengthwise and crosswise (∼20 mm × 10 mm) and peeled (epidermis removal from the abaxial side) in sterile conditions using a sharp razor, scalpel and tweezers under a Leica MZ6 stereo microscope.

### Isolation of leaf-derived protoplasts and PEG-mediated transfection

The barley protoplast isolation protocol was mainly based on the protocol for preparation of *Arabidopsis* mesophyll protoplasts (Yoo et al., 2007) with modifications described in Supplementary Method 1. Briefly, barley leaves without the epidermis were transferred into 5 mL of the enzyme solution [2% w/v Cellulase Onozuka R-10 (Duchefa, Haarlem, Netherlands), 0.1% w/v Macerozyme R-10 (Duchefa, Haarlem, Netherlands), 0.1% w/v BSA (Sigma-Aldrich, St. Louis, MO, United States), 0.55 M Mannitol (Sigma-Aldrich, St. Louis, MO, United States), pH 5.7]. Leaves were incubated in a 6-well cell culture plate (Sigma-Aldrich, St. Louis, MO, United States) in the dark for 2 h at 28°C. Released protoplasts were mixed with one volume of W5 solution [154 mM NaCl, 125 mM CaCl_2_, 5 mM KCl, 2 mM MES (Sigma-Aldrich, St. Louis, MO, United States, all), pH 5.7], filtered through a 70 μM gauze (Sigma-Aldrich, St. Louis, MO, United States), washed with 2.5 mL of W5 solution and centrifuged for 3 min at 70 × g, RT (Centrifuge 5810 R, Sigma-Aldrich, St. Louis, MO, United States). Protoplasts were gently re-suspended in 5 mL W5 solution and kept on ice for 30 min to allow cell sedimentation by gravity. Protoplasts were re-suspended to a final concentration of approximately 2 × 10^5^ protoplasts/mL in MMG solution (0.6 M Mannitol, 4 mM MES, 15 mM MgCl_2,_ pH 5.7). Fluorescein diacetate (FDA) was used to determine cell viability under a using a UV-light Zeiss Axioskop 2 Plus microscope and Canon EOS D100 digital camera.

For each transfection assay, 100 μL of protoplasts were mixed with 7 μg of construct DNA and 110 μL of freshly prepared 40% PEG-solution [0.4 M mannitol, 40% w/v PEG-4000 (Sigma-Aldrich, St. Louis, MO, United States), 0.1 M CaCl_2_ (Sigma-Aldrich, St. Louis, MO, United States), pH 5.7]. After a 10 min incubation in the dark, protoplasts were washed twice with 1.5 mL of W5 solution and centrifuged at 200 × g for 5 min (Centrifuge 5415 D, Sigma-Aldrich, St. Louis, MO, United States). Protoplasts were re-suspended in 80 μL of W5 solution and incubated in the dark for 24 h, at room temperature. Three independent transfection assays were performed per construct and averaged as biological replicates.

### *HvCslF6* promoter deletion series

The *HvCslF6* (HORVU.MOREX.r3.7HG0698110) promoter deletion constructs were generated by PCR amplification of a 3,000 bp region upstream *HvCslF6* start codon as follows: 1 μL cv. Golden Promise gDNA, 1.25 μL each primer (forward and reverse listed in Supplementary Table 7) at 10 mM, 5 μL 5X Phusion HF Buffer, 0.2 μL Phusion HF DNA polymerase (Thermo Fisher Scientific, Waltham, MA, United States) and 11.3 μL sdH_2_O in a total volume of 20 μL. Phusion PCR reaction conditions: 98°C for 30 s, 35 cycles (98°C for 10 s, 55°C for 30 s, 72°C for 30 s/kb), 72°C for 5 min and 4°C hold. Promoter fragments were ligated by NotI restriction enzyme cloning into pGreenII 0800-LUC vector which contains *luciferase* and *renilla* reporter genes, enabled to be used in Dual Luciferase Reporter Assays (Promega, Madison, WI, United States). A total of seven PCR amplicons corresponding to *HvCslF6* promoter fragments were NotI digested and purified with the QIAquick Gel Extraction Kit (Qiagen GmbH, Hilden, Germany). pGreenII-0800 vector was linearized in a similar digestion reaction using NotI with the addition of 1 μL TSAP (Promega, Madison, WI, United States) and purified as described for the *pHvCslF6* fragments. Following this, T4 DNA ligations (Promega, Madison, WI, United States) were set up for each construct and 1 μL of the ligation reaction was used for *E. coli* transformation. Plasmids were purified with QIAprep Spin Miniprep Kit (Qiagen GmbH, Hilden, Germany) and confirmed by Sanger sequencing using M13F primer at the Genome Technology facility, The James Hutton Institute.

### Dual luciferase assays

The Dual-Luciferase^®^ Reporter Assay System (Promega, Madison, WI, United States) was used to determine the promoter activity of *HvCslF6* deletion constructs in barley protoplasts, 24 h after transfection. Luminescence results were obtained using a Varioskan LUX Multimode Microplate Reader (Thermo Fisher Scientific, Waltham, MA, United States) following the dual luciferase kit instructions. Protoplasts were lysed with 80 μL of a 1X passive lysis buffer and incubated for 15 min at room temperature. In parallel, 100 μL of LAR II (Luciferase Assay Reagent II) were pre-dispensed in a Corning^®^ 96-well opaque flat bottom microplate (Thermo Fisher Scientific, Waltham, MA, United States). For all assays, 20 μL of lysed protoplasts were transferred to the 96-well plate and incubated for 1 min, room temperature. After Firefly luciferase (Fluc) activity was measured (3 s per sample with a 1.5 s delay between wells), 100 μL of Stop and Glo Reagent were added enabling Renilla luciferase (Rluc) detection (1.5 s with a 0.5 s delay between wells). For each construct tested, three technical replicates were performed per transfection assay. A negative control, which included protoplasts transformed with empty vector and dual luciferase reagents, was used in each batch of transfections. Differences in dual luciferase reporter assays across *HvCslF6* promoter deletion constructs were determined by one-way ANOVA followed by Tukey’s test using GraphPad Prism 8.4.2 Software (CA, United States). Pairwise comparisons were determined by two-tailed Student’s *t*-tests.

### R2R3-MYB phylogeny construction

The protein sequences of cv. Morex v3 genome^3^ were used to identify R2R3 MYB family members. The sequences were uploaded to a webserver^4^ for automatic identification of MYB gene family members (Pucker, 2022) using default parameters. Sequences which were not classified as R2R3 MYB were removed for the phylogenetic analysis. Protein sequences of selected *Arabidopsis* and rice MYB proteins (MYB61, including OsMYB61a and OsMYB61b and MYB46/83) were added and the sequences aligned using ClustalW in MEGA (Kumar et al., 2018). Unreliable positions in the alignment were removed using BMGE (Block Mapping and Gathering with Entropy; Criscuolo and Gribaldo, 2010) v2.0. Model selection for amino acid substitution was done in MEGA^5^ resulting in LG model plus gamma distribution with invariant sites (LG+G+I) as the best choice. The unrooted phylogenetic tree was build using Maximum Likelihood with 100 bootstrap replications to estimate bootstrap support. Only bootstrap values above 0.5 are displayed.

### Gene network construction and analysis

Gene expression values from 808 individual samples (Supplementary Table 4) were obtained and are available from Milne et al. (2021). In short, the reads were downloaded from the sequence read archive^6^ and mapped against BaRTv1 (Rapazote-Flores et al., 2019) using Salmon (Patro et al., 2017). Expression of the isoforms were added up to obtain gene expression. For network construction low expressed genes were removed by filtering for a TPM (transcript per million) of above 5 in at least a third of the samples. This condensed the number of expressed genes from 60,444 to 17,678. Weighted gene correlation network analysis (WGCNA; Langfelder and Horvath, 2008) was used for network construction. Euclidean clustering beforehand highlighted one sample from E15.MicrosporeEmbryogenic (SRR6433018) as outlier. Therefore, this sample was removed, leaving 807 samples for further analysis. The soft-thresholding power was set to 10 to achieve scale-free topology. Network analysis was carried out using the blockwise module method (Langfelder and Horvath, 2008), with a power of 10, TOMType = unsigned, corType = bicor, networkType = signed hybrid, minModuleSize = 30, maxPOutliers = 0.05, mergeCutHeight = 0.15, deepSplit = 3. Visualization of the *HvCslF6* network was performed in Gephi^7^. Size and coloring of the nodes was determined by degree centrality (the number or proportion of other nodes linked to a specific node). Edge coloring was based on weight. The subnetwork was extracted by filtering for nodes connected to *HvCslF6* (BART1_0-p53331). For this we applied an edge weight which is a threshold, whereby connections between gene represent by values greater or equal to 0.188 are correlated.

### Cloning of candidate transcription factor

A nested PCR was used to amplify *HvMYB61* (HORVU.MOREX.r3.1HG0018590) containing: 1 μL of cv. Golden Promise protoplast cDNA (1:10 diluted in sdH_2_O), 1.25 μL of each primer (forward and reverse listed on Supplementary Table 7) at 10 mM, 5 μL of 5X Phusion HF Buffer, 0.2 μL of Phusion HF DNA polymerase (Thermo Fisher Scientific, Waltham, MA, United States) and 11.3 μL of sdH_2_O in a total volume of 20 μL. Phusion PCR reaction conditions are described above. A 1 μL aliquot of the PCR mix was used as a template to attach Gateway^®^ attB sites, 8x His-tag and thrombin cleavage site at the C-terminus in a similar Phusion HF PCR reaction and gel purified. *HvMYB61* was cloned into pDONR207™, linearized with *EcoRI* by a BP reaction containing: 3 μL of the purified attB-PCR product (34 ng/μL), 1 μL of linearized pDONR207™ (50 ng/μL), 2 μL 5X BP Clonase™ Reaction Buffer (Thermo Fisher Scientific, Waltham, MA, United States) and 4 μL of TE buffer, pH 8.0 in a total volume of 10 μL. The BP reaction was incubated at RT overnight and stopped by adding 2 μL of Proteinase K and incubated for 10 min at 37°C. 1 μL of the BP reaction was used for *E. coli* transformation. Positive colonies were transferred to 4 mL of LB medium with gentamycin (100 μg/μL) and incubated overnight, 37°C and shaking at 230 rpm. Plasmids were purified using the QIAprep Spin Miniprep Kit (Qiagen GmbH, Hilden, Germany) and confirmed by Sanger sequencing. *HvMYB61* was transferred to pBract214m-HSPT plant expression vector by LR reaction containing: 2 μL of EcoRI linearized pDONR207™-HvMYB61 (40 ng/μL), 5 μL of pBract214m-HSPT (30 ng/μL), 1 μL TE Buffer, pH 8.0 and 2 μL LR Clonase™ II enzyme mix (Thermo Fisher Scientific, Waltham, MA, United States) in a total volume of 10 μL. The reaction was incubated at 25°C for 3 h and stopped by adding 1 μL of the Proteinase K (37°C for 10 min). After *E. coli* transformation, positive colonies were grown on LB medium with kanamycin (100 μg/μL) for plasmid purification. pBract214m-HSPT-HvMYB61 construct sequence was confirmed as described above using the ZmUbi forward primer (Supplementary Table 7).

### RNA isolation, cDNA synthesis, and quantitative real-time PCR on protoplasts

Total mRNA was extracted from ∼2 × 10^5^ protoplasts (100 μL) using TRIzol^®^ Reagent (Thermo Fisher Scientific, Waltham, MA, United States) according to the manufacturer’s instructions. RNA concentration and purity were measured using NanoDrop 2000 (Thermo Fisher Scientific, Waltham, MA, United States). Total mRNA to cDNA conversion was performed using cDNA EcoDry™ Premix (Takara, Kyoto, Japan). For each reaction, 100 ng of total mRNA was added to the lyophilized master mix following the manufacturer’s instructions. Quantitative real-time PCR (qRT-PCR) was performed in a StepOne Real-Time PCR machine (Thermo Fisher Scientific, Waltham, MA, United States) using PowerUp SYBR Green Master Mix (Thermo Fisher Scientific, Waltham, MA, United States) to determine *HvCslF6* relative transcript abundance. Three independent experiments were performed, each of them included: HvMYB61 PEG-mediated transfection, RNA extraction, cDNA synthesis and qPCRs, respectively. Three replicate qRT-PCR reactions were performed for each cDNA sample (technical replicates) including a negative control (sdH_2_O as template). Each qRT-PCR reaction contained: 2 μL protoplast cDNA (1:2 dilution), 5 μL SYBR Green, 1 μL each forward and reverse primer at 4 mM and 1 μL of sdH_2_O in a total volume of 10 μL. Primer sequences for *HvCslF6* and qRT-PCR conditions were used as in Burton et al. (2008) and described in Supplementary Table 7. Relative *HvCslF6* gene expression was normalized to α-*Tubulin*, *Gapdh* and *Hsp70* housekeeping genes and calculated using the 2^–ΔΔ*CT*^ method (Vandesompele et al., 2002) for multiple control genes. Statistical differences in *HvCslF6* expression were determined by Student’s *t*-test compared to WT protoplasts (empty vector) using GraphPad Prism 9.1 Software (CA, United States).

## Data availability statement

The raw data supporting the conclusions of this article will be made available by the authors, without undue reservation.

## Author contributions

GG-G, GD, KH, MT, RB, and RW conceived the work. GD initiated the cloning of *HvCslF6* promoter deletion series. GG-G performed the laboratory work, TFBS, and data analyses. KH carried out co-expression analyses. MS performed the gene network construction. RS carried out the barley transformations. GG-G, MS, KH, and MT drafted the manuscript. GD, GF, RB, and RW reviewed the manuscript. All authors have read and approved the manuscript.

## Footnotes

1
https://wheat.pw.usda.gov/blast/

2
http://plants.ensembl.org

3
https://plants.ensembl.org/Hordeum_vulgare/Info/Index

4
http://pbb.bot.nat.tu-bs.de/MYB_annotator

5
https://www.megasoftware.net/

6
https://www.ncbi.nlm.nih.gov/sra

7
https://gephi.org/
