# Supplementary material for: Identification of candidate MYB transcription factors that influence CslF6 expression in barley grain
Source: Front Plant Sci. 2022 Sep 8;13:883139. doi: 10.3389/fpls.2022.883139 (PMC9493323; doi:10.3389/fpls.2022.883139)
Supplement: Supplementary file 1 [file Data_Sheet_1.PDF]

Isolation & PEG-Mediated Transfection of Barley Leaf-Derived Protoplasts

Reagents and solutions

Enzyme solution for barley (stored at -20°C)

2 % w/v Cellulase R-10 (Duchefa)
0.1 % w/v Macerozyme R-10 (Duchefa)
0.1 % w/v BSA
0.55 M Mannitol
pH 5.7
Sterilize by filtration 0.2 µM

W5 sol (stored RT)

154 mM NaCl
125 mM CaCl2
5 mM KCl
2 mM MES
pH 5.7
Sterilize by filtration 0.2 µM

MMG sol (stored RT)

4 mM MES
0.6 M Mannitol
15 mM MgCl2
pH 5.7
Sterilize by filtration 0.2 µM

40% PEG sol

(Freshly made while protoplasts are in step #3)

40% w/v PEG-4000
0.4M Mannitol
0.1M CaCl2

Adjust pH to 5.7–5.9
Adjust volume with sdH_2O and sterilize by filtration 0.2 µM.

WI sol (stored RT) – optional solution

0.5 M Mannitol
20 mM KCL
4 mM MES
pH 5.7
Sterilize by filtration 0.2 µM

Comments:
Enzyme solution (e.g. 25 mL sol); In a sterile beaker, weigh 0.50 g of cellulase (Cellulase R-10, Duchefa), 25 mg BSA and 25 mg of Macerozyme R-10. Using Pectinase (Y23) instead of Macerozyme R-10 yielded good results too. Add 20 mL 0.55M mannitol and stir at RT until the contents are dissolved. Adjust pH to 5.9 with 0.1M KOH. Check final vol (25 mL). Sterilize by filtration (0.2 µM). Make 5 mL aliquots and store to up 6 months at -20°C.

W5 solution was usually prepared in 100 mL (total volume).

MMG solution was prepared in 15 mL (total volume).

40% PEG solution was freshly prepared in 5 mL (total volume).

Protocol

Isolation of leaf-derived barley protoplasts **Estimated time: 4,5 h**

1. Harvest 0.25 g of 3-week (15 to 20 days) old barley leaves (Golden Promise *cv*).
2. *In the laminar flow cabinet*: gently remove the epidermis (with a sharp razor and tweezers) from primary leaves lengthwise. Discard the epidermis.
3. Transfer peeled material into 5 mL enzyme solution (small Petri dish/6-well plate) and incubate 2h at 28°C, no shaking.

   Completely digested material will have a transparent appearance.
4. Add 1 volume of W5 sol (5 mL) to dilute the enzyme solution, making protoplast collection easier.
5. Take another Petri dish and decant the solution through a 70 µM cell strainer using a plastic Pasteur pipette.
6. Pre-dispense 2.5 mL of W5 solution to a sterile 15/30 mL round-bottom Falcon tube.
7. Carefully transfer the filtrated protoplast suspension solution to the Falcon tube containing 2.5 mL of W5 solution using a Pasteur pipette.
8. Centrifuge for 3 min, at 70 x g, RT (swinging bucket).

   Handle protoplasts carefully
9. Protoplasts will form a pellet although it is possible that some will remain in the supernatant. Discard supernatant.
10. Re-suspend protoplasts in 5 mL W5 solution and leave on ice for 30 min. Use this time to estimate the protoplast concentration using a haemocytometer/Neubauer chamber (Step 13).

    Protoplasts will sediment at the bottom by gravity
11. Re-suspended protoplasts in MMG solution. Calculate the amount of MMG solution to add (~2 x 10^5 protoplasts for transient assays).
12. Protoplast viability determination. Mix an aliquot of protoplasts (10 µL) in MMG solution with 1 µL of fluorescein diacetate (FDA) and incubate for 1 min.
13. Transfer 10 µL of the mix into a haemocytometer/ Neubauer chamber to determine protoplast yield (Protoplasts/mL= average number of cells x dilution factor x 10^4. Calculations based on Neubauer chamber with 0.1 cm x 0.1 cm x 0.01 cm dimensions). View under a microscope equipped with a UV-light source (GFP filter).

    Live protoplasts will fluoresce in bright green/ dead cells and debris in red.
14. Keep protoplasts on ice.

Proceed to PEG-mediated inoculation immediately

<u>PEG-mediated inoculation of protoplasts</u> **Estimated time: 1.5 h for 6 constructs**

1. In a round bottom Falcon tube or 2 mL Eppendorf tube. Add ~2 x 10^5 of protoplasts and mix gently. (Usually 100 µL yields ~2x 10^5 protoplasts, otherwise calculate dilution).
2. Add 5 µg – 10 µg of plasmid DNA.
   5–7 µg *of plasmid DNA yielded good results for YFP/GFP-expressing constructs.*
3. Add 110 µL of fresh 40% PEG solution pH 5.7–5.9, then mix completely by gently tapping the tube.
4. Incubate the transfection mixture at RT for 10 min in the dark.
5. Dilute the transfection mixture with 1.5 mL W5 solution and mix well by gently inverting the tube to stop transfection process.
6. Pellet protoplasts by centrifugation at 200 x g for 5 min and remove supernatant, always keep a ~100 µL solution volume to avoid protoplast to dry.
7. Repeat wash (step 5)
8. Re-suspend protoplasts gently in 80 µL W5 solution (also possible to re-suspend in mannitol-based WI solution). Protoplasts were viable up to 3 days in W5 solution.
9. Incubate in the dark for 16-24 h at RT. Incubation time depends on reporter gene used/assay.

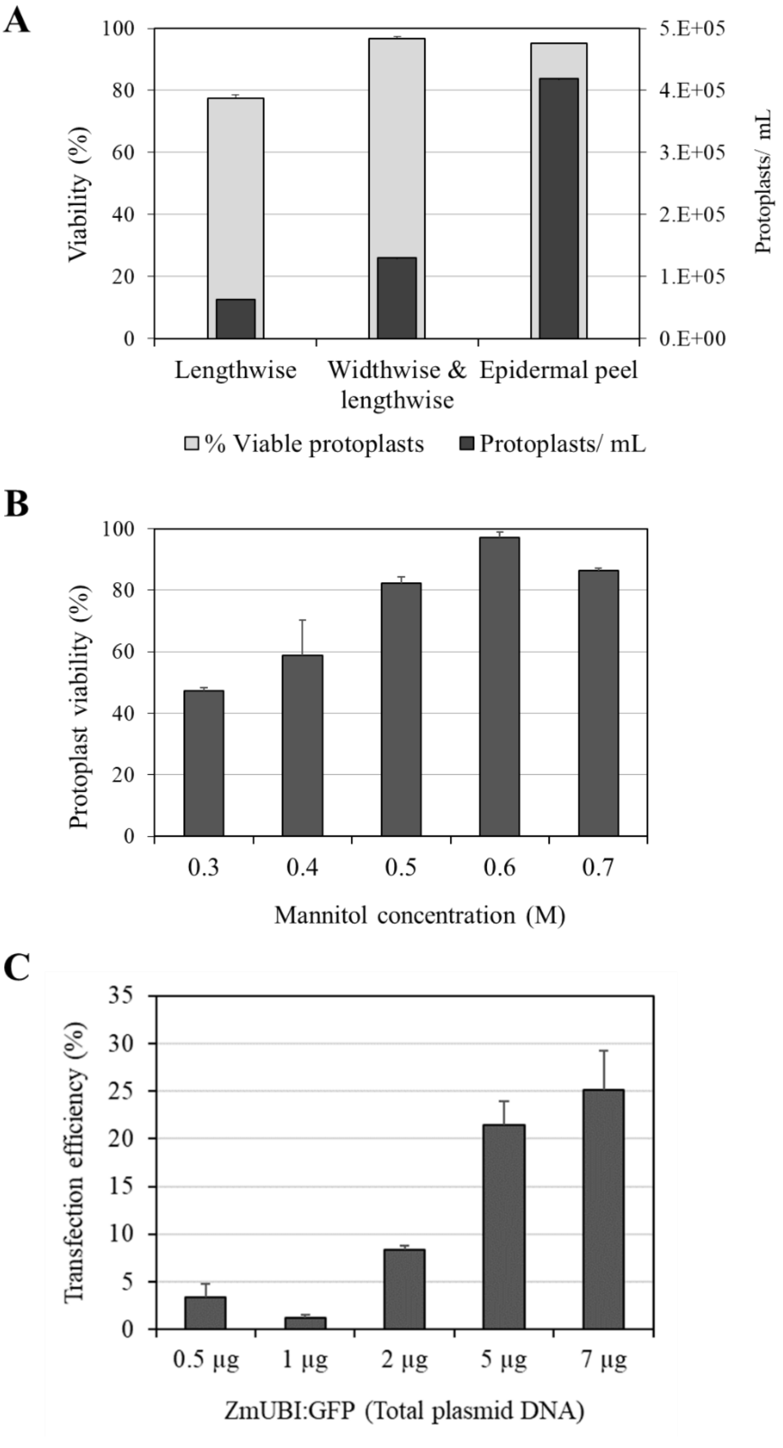

Figure 1. Optimisation of protoplast isolation and transfection assays. A) Protoplast viability (%) and cell concentration (protoplasts/ mL) results depending on leaf tissue preparation. Cell counting was averaged from three microscope fields per assay method using a Neubauer chamber. B) Effect of mannitol concentration (M) on protoplast viability (%). Fluorescein diacetate (FDA) was used to determine cell viability. Protoplasts were screened under a Zeiss Axioskop 2 Plus microscope with a UV light source (GFP filter). C) Effect of DNA concentration (µg) on transfection efficiency of barley protoplasts using ZmUBI:bcoGFP vector. Error bars represent standard error associated to three experimental replicates per treatment (n=3).

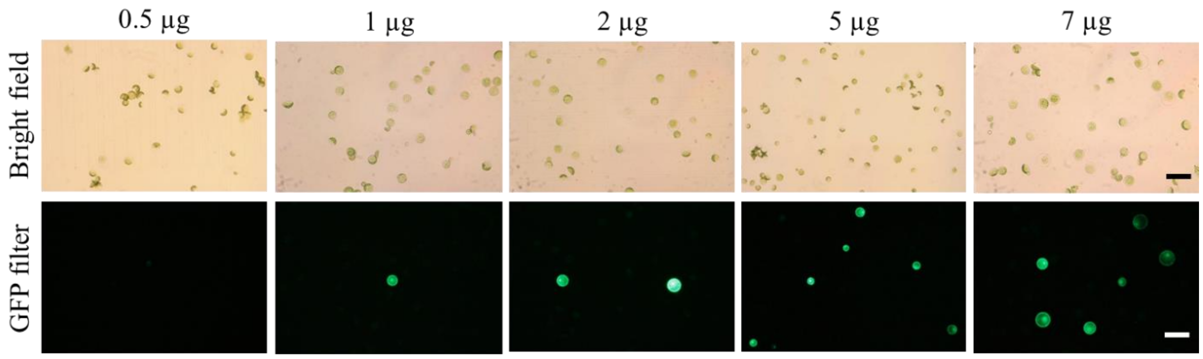

Figure 2. Fluorescence microscope images (10X) of barley leaf-derived protoplasts transfected with ZmUBI:bcoGFP vector. Top row, bright field; below GFP-filtered images. GFP was excitated at 488 nm and emission collected from 500 to 530 nm. Scale bars are 100 µm.

References

Bai, Y., Han, N., Wu, J., Yang, Y., Wang, J., Zhu, M., & Bian, H. (2014). A transient gene expression system using barley protoplasts to evaluate microRNAs for post-transcriptional regulation of their target genes. Plant Cell, Tissue and Organ Culture, 119(1), 211-219.

Rao A. L. N. (2007) Preparation and inoculation of mesophyll protoplasts from monocotyledenous and dicotyledenous hosts. Current Protocols in Microbiology 4, 16D.2.1-16D.2.8.

Yoo, S. D., Cho, Y. H., & Sheen, J. (2007). Arabidopsis mesophyll protoplasts: a versatile cell system for transient gene expression analysis. Nature protocols, 2(7), 1565-1572.
