# Supplementary material for: Identification of candidate MYB transcription factors that influence CslF6 expression in barley grain
Source: Front Plant Sci. 2022 Sep 8;13:883139. doi: 10.3389/fpls.2022.883139 (PMC9493323; doi:10.3389/fpls.2022.883139)
Supplement: Supplementary file 2 [file Data_Sheet_2.PDF]

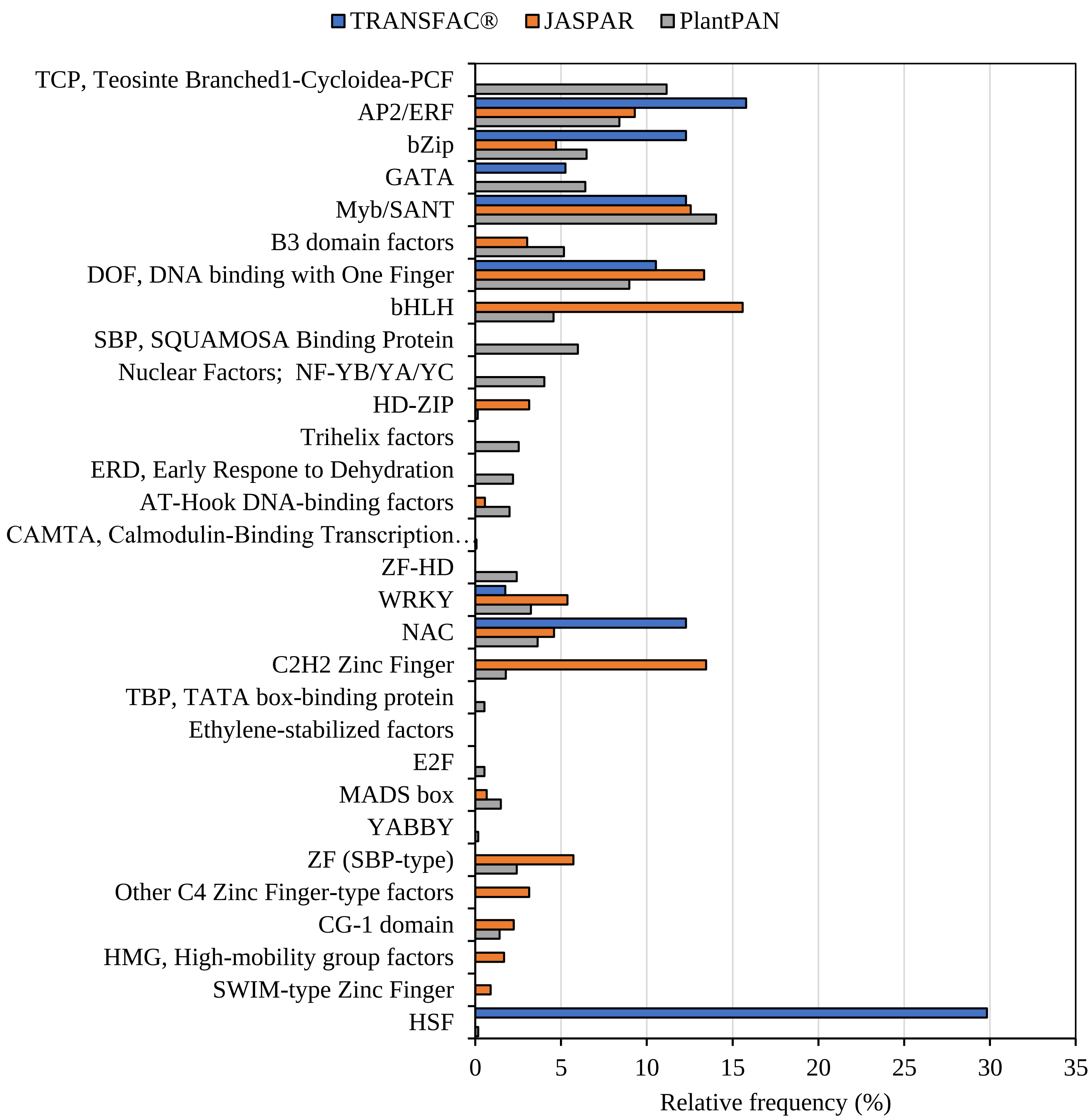

Figure S1. Relative frequency of predicted transcription factor binding sites (TFBSs), grouped in TF classes in a -3,000 bp *HvCslF6* upstream region predicted by three different in silico tools: TRANSFAC® [v2014; Matys *et al*., (2006)], JASPAR [v2020; Fornes *et al*., (2020)] and PlantPAN [v3.0; Chow *et al*., (2019)]. Predicted TFBSs with an absolute frequency of ≤5 were omitted from this graph for display purposes (This included:14 low-represented predicted motifs identified by TRANSFAC®, 27 by JASPAR and 40 by PlantPAN).

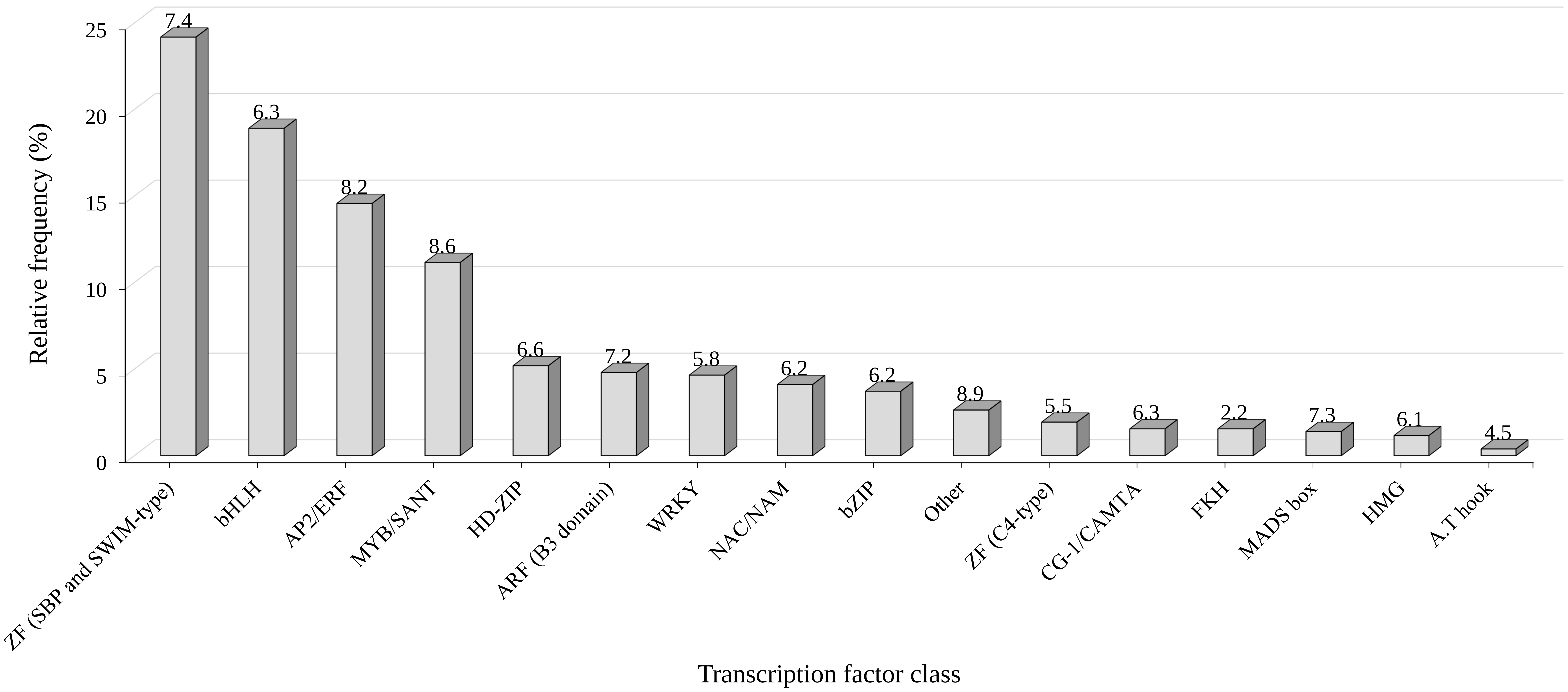

Figure S2. Relative frequency (%) of predicted TFBSs (grouped in TF classes) identified within a -1,000 bp *HvCslF6* promoter region using JASPAR's unfiltered output (Fornes *et al*., 2020). TF class "other" indicate a mixed class of low-represented TFBSs (frequency within *HvCslF6* full length promoter ≤ 2%). Average scores for each TF class were inferred from position frequency matrices (PFMs) and TF flexible models (TFFMs) in JASPAR and are shown on top of each column.

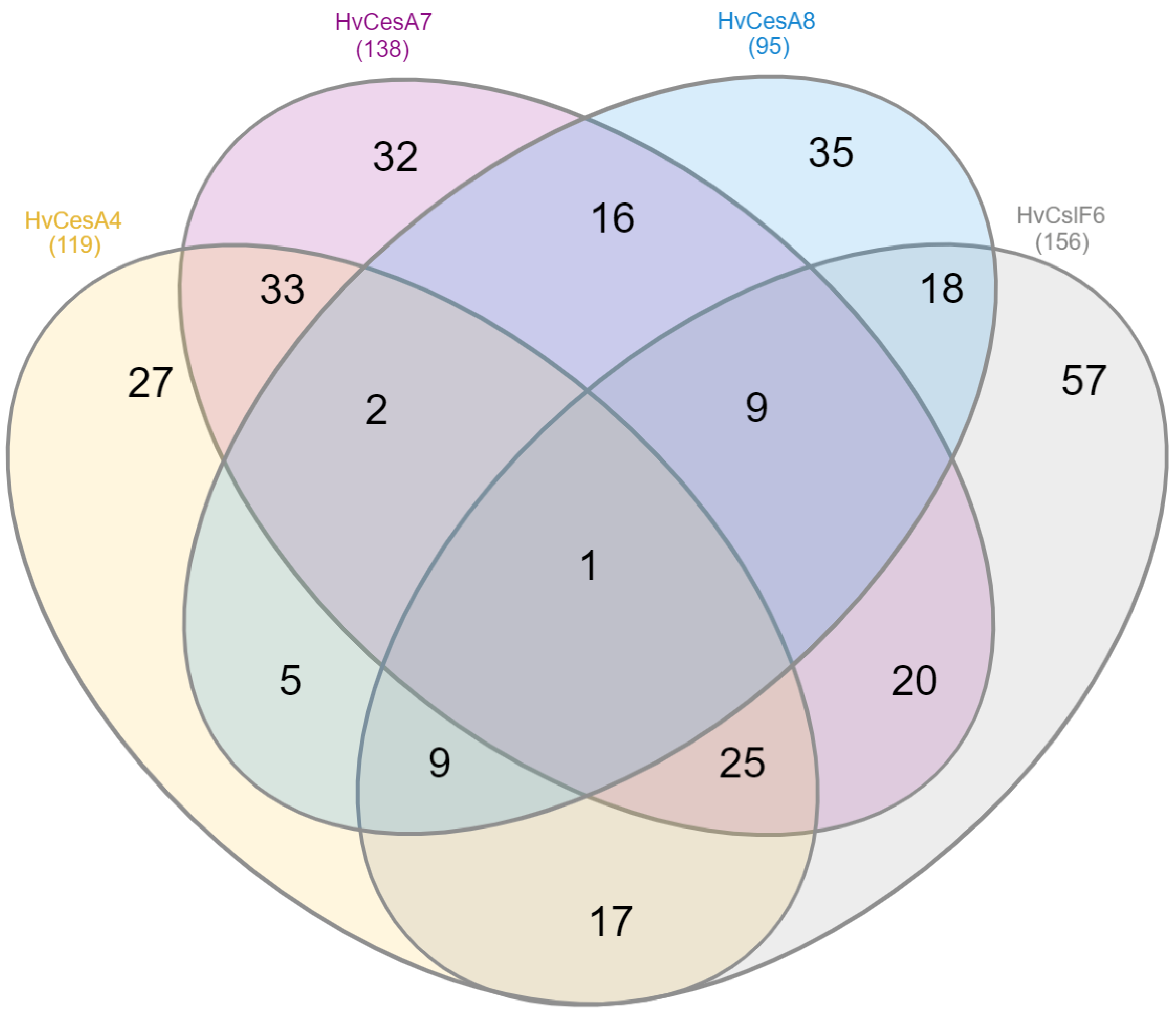

Figure S3. Venn diagram of TFBSs predicted within *HvCesA4*, *HvCesA7*, *HvCesA8* and *HvCslF6* putative promoter regions (-1,000 bp) with identical filtering parameters as described in Fig 2. SQUAMOSA promoter binding protein-like (SBP or SPL) was the only TFBS found in common. Venn diagram was generated using InteractiVenn (Heberle *et al*., 2015).

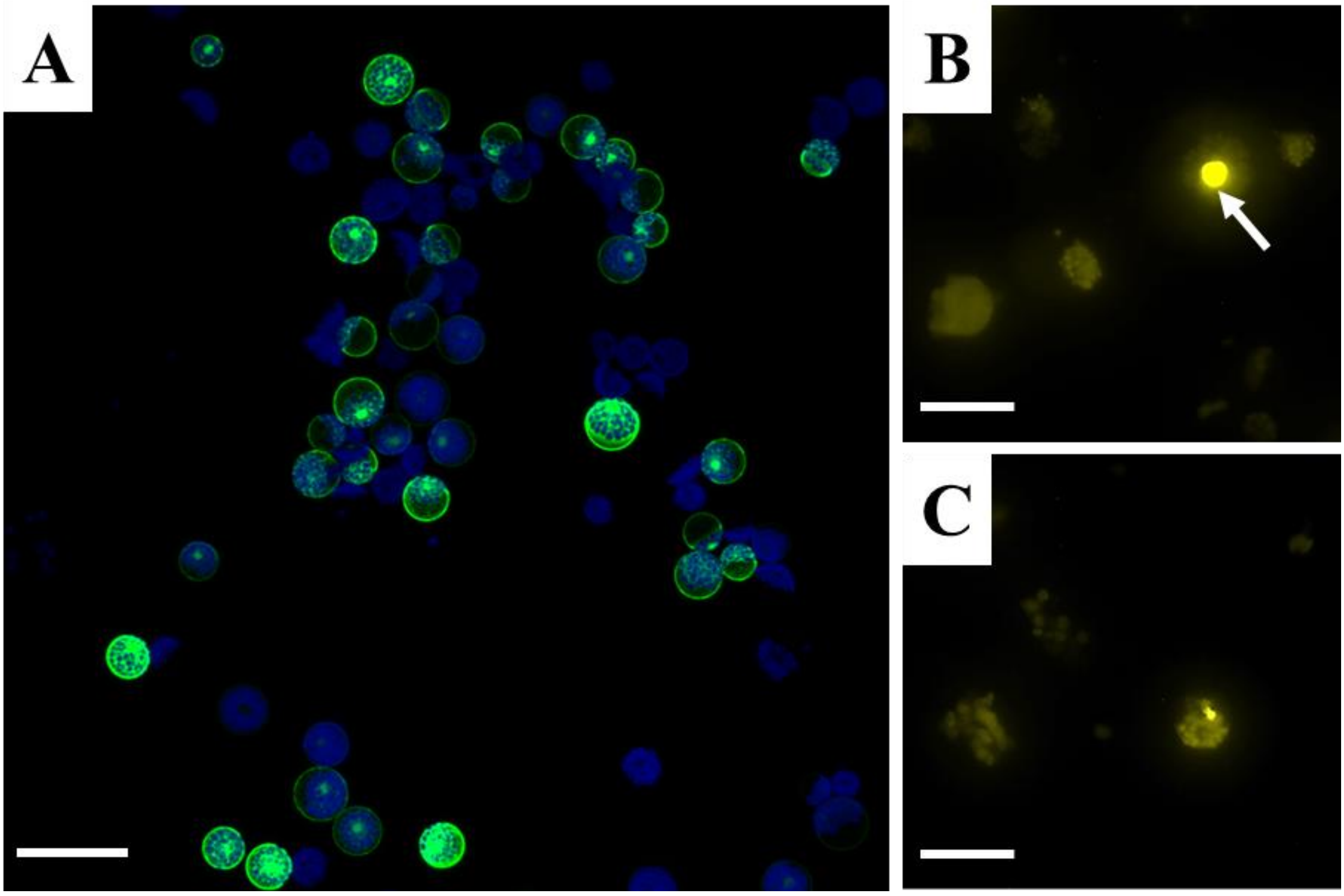

Figure S4. Images of barley protoplasts 24 h after being transfected with A) pZmUBI:bcoGFP-ER, barley codon optimised. Confocal microscope 10X, scale bar is 100 µm. GFP was excitated at 488 nm and emission collected from 499 to 530 nm, in green. Autofluorescence, typically from chloroplasts was detected from 650 to 690 nm. B) pOsAct:3nlsYFP C) pHvCslF6:nlsYFP (3,000 bp). Both B) and C) are fluorescence microscope images, 20X. YFP was excitated at 561 nm and emission collected from 500 to 530 nm, in yellow. Scale bars are 20 µm.

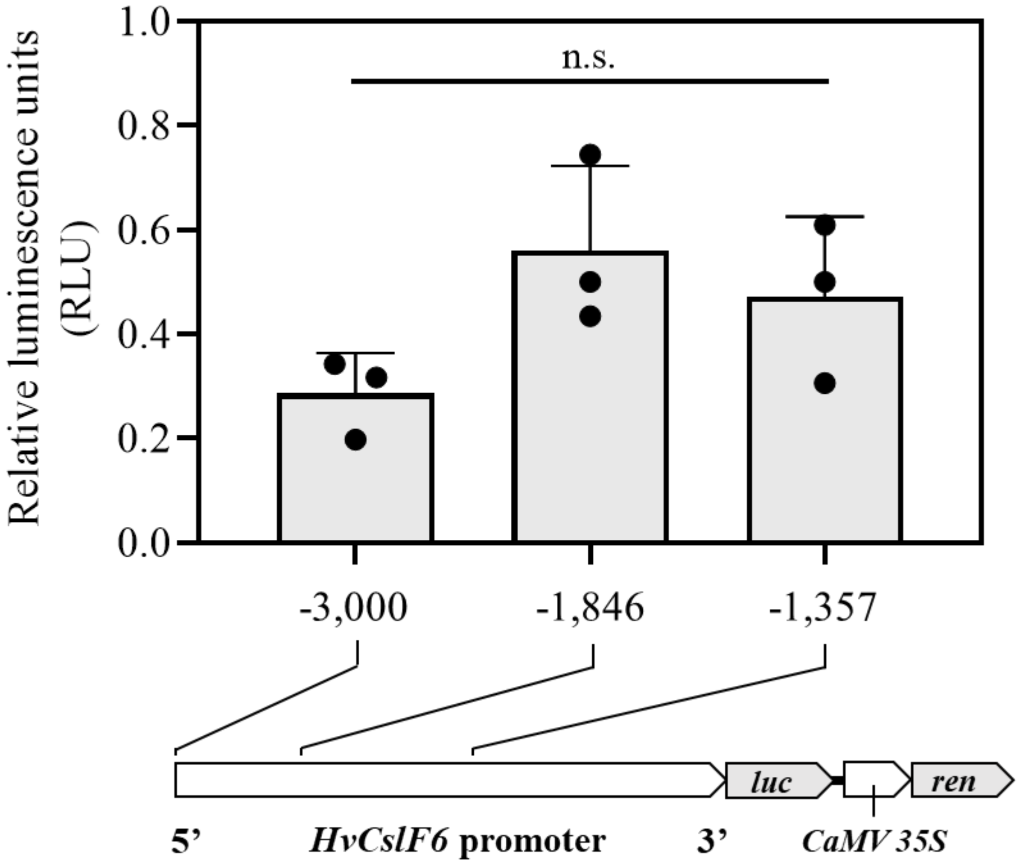

Figure S5. Dual luciferase reporter activities expressed as luciferase/renilla ratio of 5' *HvCslF6* promoter deletion constructs from -3,000 bp, -1,846 bp and -1,357 bp in barley protoplasts. Three independent transfection assays were performed per construct and averaged to calculate mean reporter activity. Three negative controls (empty vector lacking *HvCslF6* promoter) were performed in parallel and luciferase background activity subtracted from tested constructs. No significant differences were observed across the tested constructs ($p = 0.665$, one-way ANOVA).

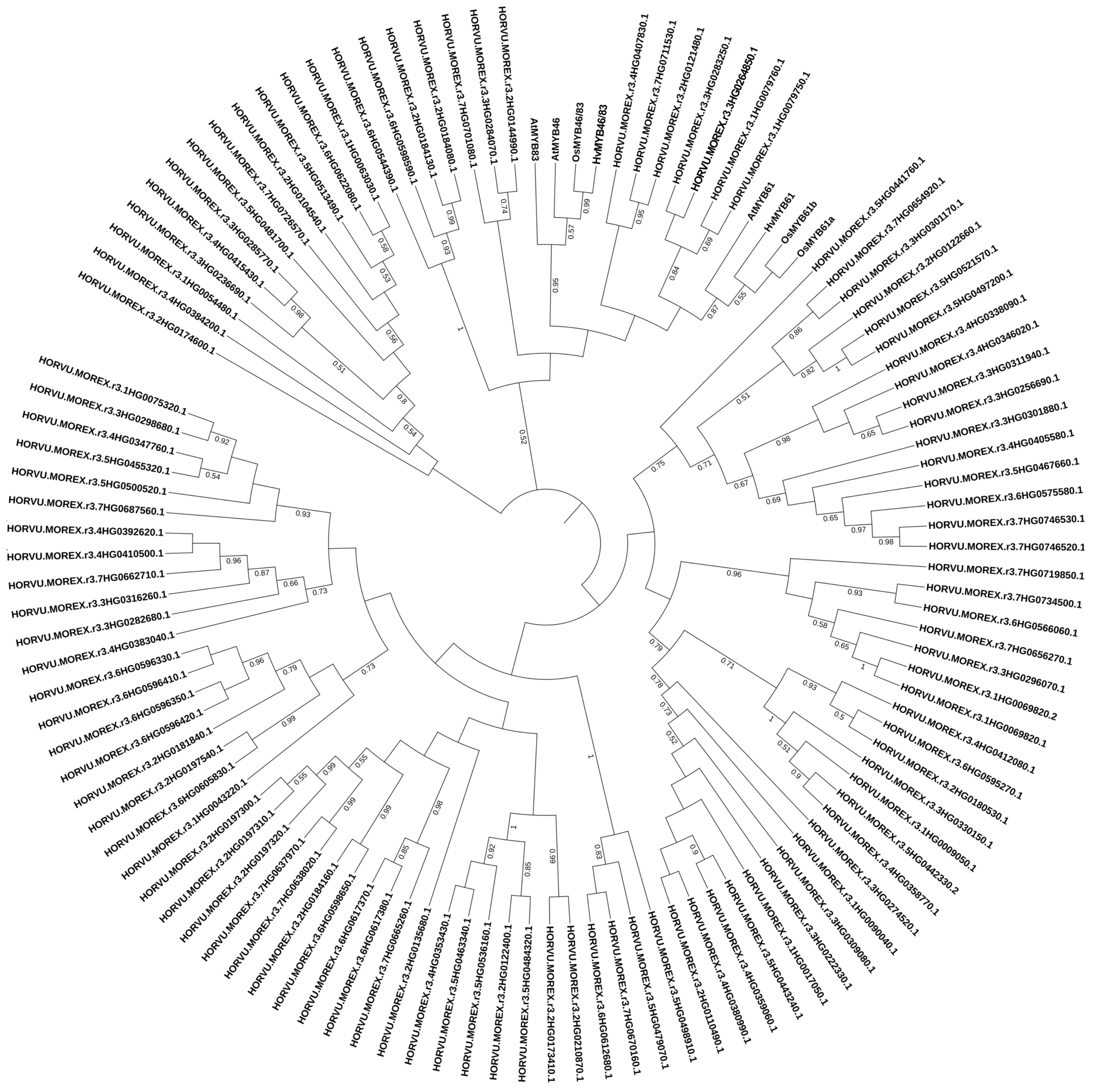
HORVU.MOREX.r3.2HG0174600.1
HORVU.MOREX.r3.4HG0384200.1
HORVU.MOREX.r3.1HG0054480.1
HORVU.MOREX.r3.3HG0236690.1
HORVU.MOREX.r3.4HG0415430.1
HORVU.MOREX.r3.3HG0285770.1
HORVU.MOREX.r3.5HG0481700.1
HORVU.MOREX.r3.7HG0726570.1
HORVU.MOREX.r3.2HG0104540.1
HORVU.MOREX.r3.5HG0513490.1
HORVU.MOREX.r3.6HG0622080.1
HORVU.MOREX.r3.1HG0063030.1
HORVU.MOREX.r3.6HG0544390.1
HORVU.MOREX.r3.6HG0598590.1
HORVU.MOREX.r3.2HG0184130.1
HORVU.MOREX.r3.2HG0184080.1
HORVU.MOREX.r3.7HG0701080.1
HORVU.MOREX.r3.3HG0284070.1
HORVU.MOREX.r3.2HG0144990.1
AtMYB83
AtMYB46
OsMYB46/83
HvMYB46/83
HORVU.MOREX.r3.4HG0407830.1
HORVU.MOREX.r3.7HG0711530.1
HORVU.MOREX.r3.2HG0121480.1
HORVU.MOREX.r3.3HG0283250.1
HORVU.MOREX.r3.3HG0264850.1
HORVU.MOREX.r3.1HG0079760.1
HORVU.MOREX.r3.1HG0079750.1
AtMYB61
HvMYB61
OsMYB61b
OsMYB61a
HORVU.MOREX.r3.5HG0441760.1
HORVU.MOREX.r3.7HG0654920.1
HORVU.MOREX.r3.3HG0301170.1
HORVU.MOREX.r3.2HG0122660.1
HORVU.MOREX.r3.5HG0521570.1
HORVU.MOREX.r3.5HG0497200.1
HORVU.MOREX.r3.4HG0338090.1
HORVU.MOREX.r3.4HG0346020.1
HORVU.MOREX.r3.3HG0311940.1
HORVU.MOREX.r3.3HG0256690.1
HORVU.MOREX.r3.3HG0301880.1
HORVU.MOREX.r3.4HG0405580.1
HORVU.MOREX.r3.5HG0467660.1
HORVU.MOREX.r3.6HG0575580.1
HORVU.MOREX.r3.7HG0746530.1
HORVU.MOREX.r3.7HG0746520.1
HORVU.MOREX.r3.7HG0719850.1
HORVU.MOREX.r3.7HG0734500.1
HORVU.MOREX.r3.6HG0566060.1
HORVU.MOREX.r3.7HG0656270.1
HORVU.MOREX.r3.3HG0296070.1
HORVU.MOREX.r3.1HG0069820.2
HORVU.MOREX.r3.1HG0069820.1
HORVU.MOREX.r3.4HG0412080.1
HORVU.MOREX.r3.6HG0595270.1
HORVU.MOREX.r3.2HG0180530.1
HORVU.MOREX.r3.3HG0330150.1
HORVU.MOREX.r3.1HG0009050.1
HORVU.MOREX.r3.5HG0442330.2
HORVU.MOREX.r3.4HG0358770.1
HORVU.MOREX.r3.3HG0274520.1
HORVU.MOREX.r3.1HG0090040.1
HORVU.MOREX.r3.3HG0309080.1
HORVU.MOREX.r3.3HG0222330.1
HORVU.MOREX.r3.1HG0017050.1
HORVU.MOREX.r3.5HG0443240.1
HORVU.MOREX.r3.4HG0359060.1
HORVU.MOREX.r3.4HG0380990.1
HORVU.MOREX.r3.2HG0110490.1
HORVU.MOREX.r3.5HG0498910.1
HORVU.MOREX.r3.5HG0479070.1
HORVU.MOREX.r3.7HG0670160.1
HORVU.MOREX.r3.6HG0612680.1
HORVU.MOREX.r3.2HG0210870.1
HORVU.MOREX.r3.2HG0173410.1
HORVU.MOREX.r3.5HG0484320.1
HORVU.MOREX.r3.2HG0122400.1
HORVU.MOREX.r3.5HG0536160.1
HORVU.MOREX.r3.5HG0463340.1
HORVU.MOREX.r3.4HG0353430.1
HORVU.MOREX.r3.2HG0135680.1
HORVU.MOREX.r3.7HG0665260.1
HORVU.MOREX.r3.6HG0617380.1
HORVU.MOREX.r3.6HG0617370.1
HORVU.MOREX.r3.6HG0598650.1
HORVU.MOREX.r3.2HG0184160.1
HORVU.MOREX.r3.7HG0638020.1
HORVU.MOREX.r3.7HG0637970.1
HORVU.MOREX.r3.2HG0197320.1
HORVU.MOREX.r3.2HG0197310.1
HORVU.MOREX.r3.2HG0197300.1
HORVU.MOREX.r3.1HG0043220.1
HORVU.MOREX.r3.6HG0605830.1
HORVU.MOREX.r3.2HG0197540.1
HORVU.MOREX.r3.2HG0181840.1
HORVU.MOREX.r3.6HG0596420.1
HORVU.MOREX.r3.6HG0596350.1
HORVU.MOREX.r3.6HG0596410.1
HORVU.MOREX.r3.6HG0596330.1
HORVU.MOREX.r3.4HG0383040.1
HORVU.MOREX.r3.3HG0282680.1
HORVU.MOREX.r3.3HG0316260.1
HORVU.MOREX.r3.7HG0662710.1
HORVU.MOREX.r3.4HG0410500.1
HORVU.MOREX.r3.4HG0392620.1
HORVU.MOREX.r3.7HG0687560.1
HORVU.MOREX.r3.5HG0500520.1
HORVU.MOREX.r3.5HG0455320.1
HORVU.MOREX.r3.4HG0347760.1
HORVU.MOREX.r3.3HG0298680.1
HORVU.MOREX.r3.1HG0075320.1

Figure S6. The protein sequences of cv. Morex v3 genome (Available from: https://plants.ensembl.org/Hordeum_vulgare/Info/Index) were used to identify R2R3 MYB family members. The sequences were uploaded to a webserver (http://pbb.bot.nat.tu-bs.de/MYB_annotator) for automatic identification of MYB gene family members (Pucker, 2022) using default parameters. Sequences which were not classified as R2R3 MYB were removed for the phylogenetic analysis. Protein sequences of selected *Arabidopsis* and rice MYB proteins (MYB61, MYB46 and MYB83) were added, and the sequences aligned using ClustalW in MEGA (Kumar *et al.*, 2018). Unreliable positions in the alignment were removed using BMGE (Block Mapping and Gathering with Entropy; Criscuolo and Gribaldo, 2010) v2.0. Model selection for amino acid substitution was done in MEGA (Available from: https://www.megasoftware.net/) resulting in LG model plus gamma distribution with invariant sites (LG+G+I) as the best choice. The unrooted phylogenetic tree was build using Maximum Likelihood with 100 bootstrap replications to estimate bootstrap support. Only bootstrap values above 0.5 are displayed.

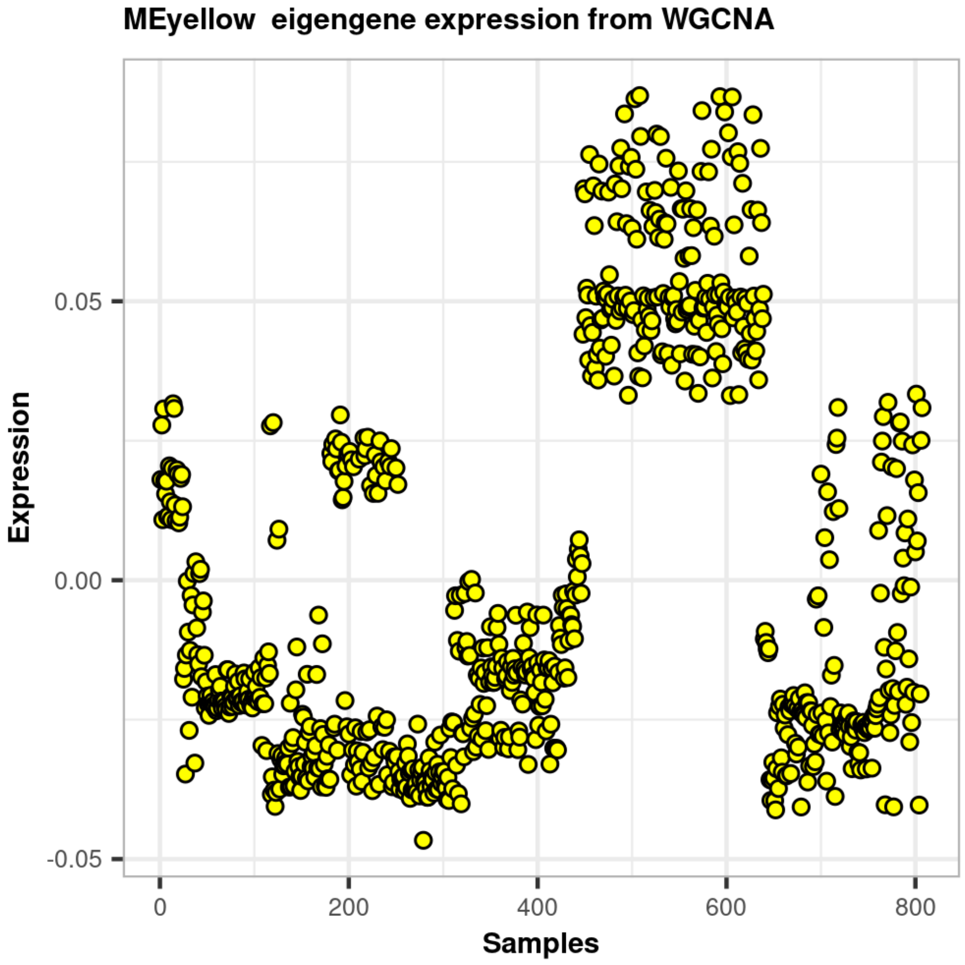

Figure S7. Relative gene expression of the yellow module across all 807 samples. Samples used for the gene network analysis with project description number and identification number from the sequence read archive (SRA) are described in Table S7.

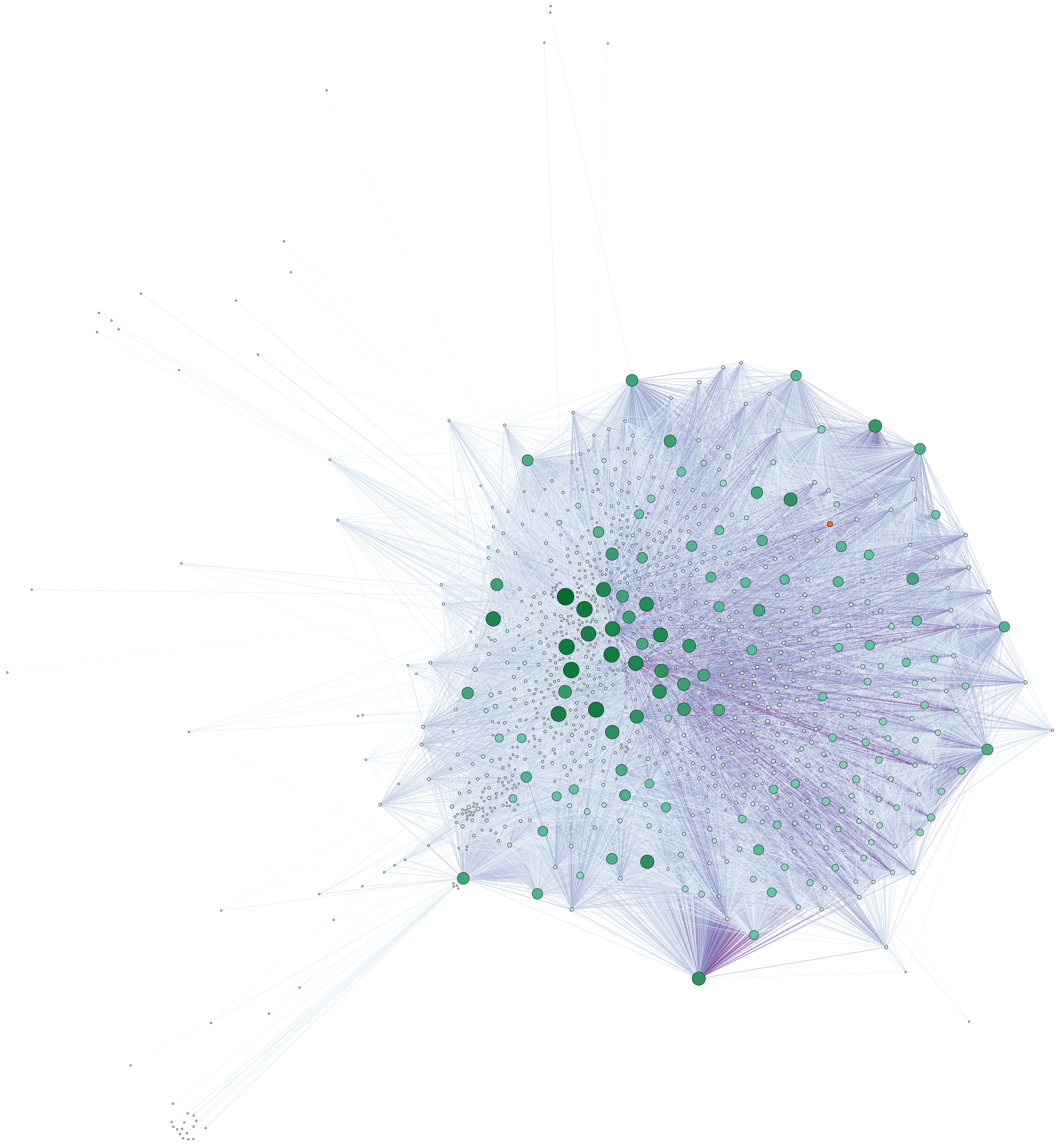

Figure S8. Yellow module network, visualised using Gephi (https://gephi.org/). Node size and colour correspond to gene degree centrality. Big and dark green nodes have a high number of linked nodes, while small and light green nodes only have a small number of linked nodes. The edges are coloured by weight. A dark purple corresponds to a high weight and therefore correlation, while a light purple colour corresponds to a lower weight and less strong correlation between the nodes. *HvCslF6* is highlighted in orange.

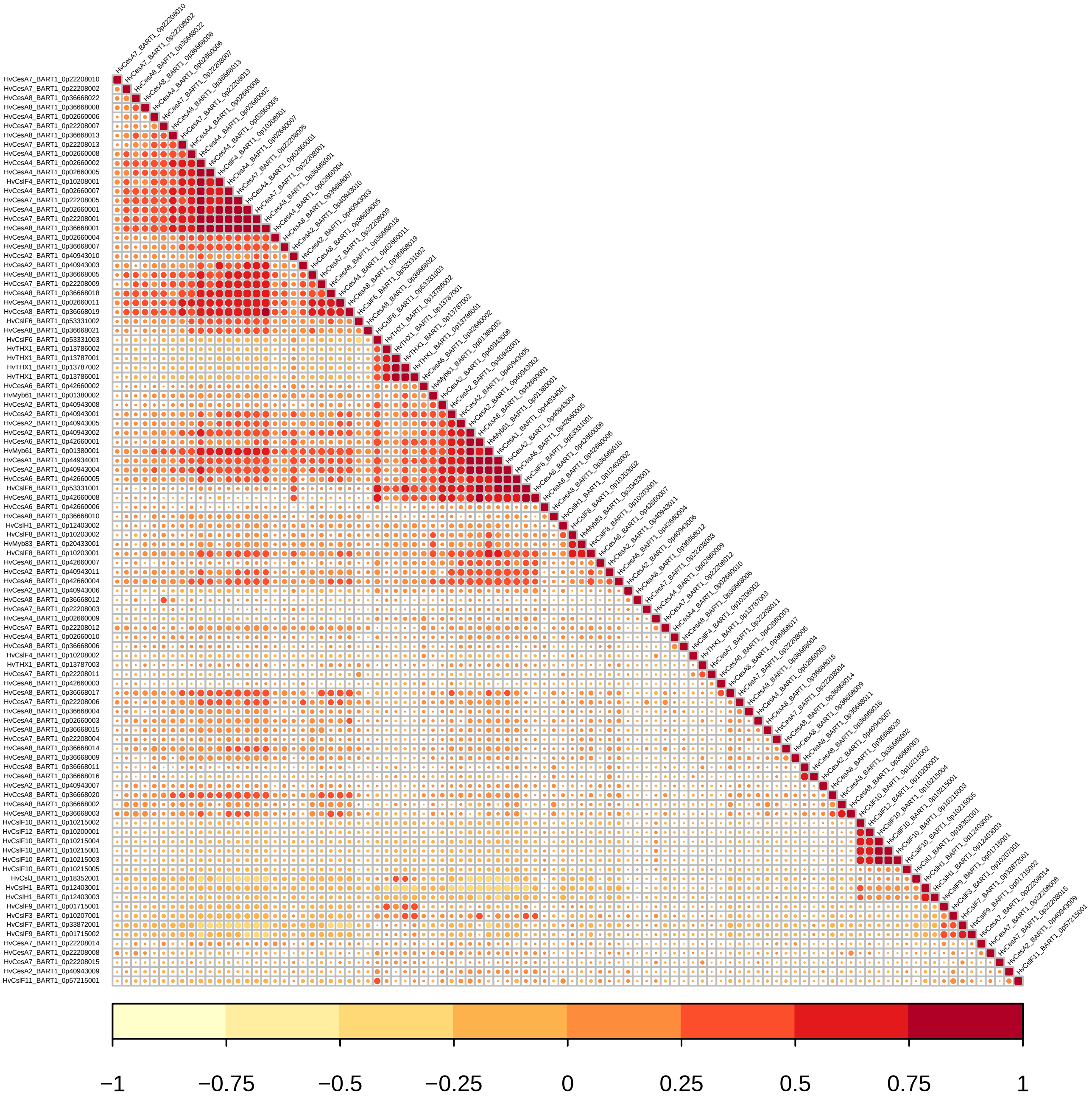
HvCesA7_BART1_0p22208010
HvCesA7_BART1_0p22208002
HvCesA8_BART1_0p36668022
HvCesA8_BART1_0p36668008
HvCesA4_BART1_0p02660006
HvCesA7_BART1_0p22208007
HvCesA8_BART1_0p36668013
HvCesA7_BART1_0p22208013
HvCesA4_BART1_0p02660008
HvCesA4_BART1_0p02660002
HvCesA4_BART1_0p02660005
HvCslF4_BART1_0p10208001
HvCesA4_BART1_0p02660007
HvCesA7_BART1_0p22208005
HvCesA4_BART1_0p02660001
HvCesA7_BART1_0p22208001
HvCesA8_BART1_0p36668001
HvCesA4_BART1_0p02660004
HvCesA8_BART1_0p36668007
HvCesA2_BART1_0p40943010
HvCesA2_BART1_0p40943003
HvCesA8_BART1_0p36668005
HvCesA7_BART1_0p22208009
HvCesA8_BART1_0p36668018
HvCesA4_BART1_0p02660011
HvCesA8_BART1_0p36668019
HvCslF6_BART1_0p53331002
HvCesA8_BART1_0p36668021
HvCslF6_BART1_0p53331003
HvTHX1_BART1_0p13786002
HvTHX1_BART1_0p13787001
HvTHX1_BART1_0p13787002
HvTHX1_BART1_0p13786001
HvCesA6_BART1_0p42660002
HvMyb61_BART1_0p01380002
HvCesA2_BART1_0p40943008
HvCesA2_BART1_0p40943001
HvCesA2_BART1_0p40943005
HvCesA2_BART1_0p40943002
HvCesA6_BART1_0p42660001
HvMyb61_BART1_0p01380001
HvCesA1_BART1_0p44934001
HvCesA2_BART1_0p40943004
HvCesA6_BART1_0p42660005
HvCslF6_BART1_0p53331001
HvCesA6_BART1_0p42660008
HvCesA6_BART1_0p42660006
HvCesA8_BART1_0p36668010
HvCslH1_BART1_0p12403002
HvCslF8_BART1_0p10203002
HvMyb83_BART1_0p20433001
HvCslF8_BART1_0p10203001
HvCesA6_BART1_0p42660007
HvCesA2_BART1_0p40943011
HvCesA6_BART1_0p42660004
HvCesA2_BART1_0p40943006
HvCesA8_BART1_0p36668012
HvCesA7_BART1_0p22208003
HvCesA4_BART1_0p02660009
HvCesA7_BART1_0p22208012
HvCesA4_BART1_0p02660010
HvCesA8_BART1_0p36668006
HvCslF4_BART1_0p10208002
HvTHX1_BART1_0p13787003
HvCesA7_BART1_0p22208011
HvCesA6_BART1_0p42660003
HvCesA8_BART1_0p36668017
HvCesA7_BART1_0p22208006
HvCesA8_BART1_0p36668004
HvCesA4_BART1_0p02660003
HvCesA8_BART1_0p36668015
HvCesA7_BART1_0p22208004
HvCesA8_BART1_0p36668014
HvCesA8_BART1_0p36668009
HvCesA8_BART1_0p36668011
HvCesA8_BART1_0p36668016
HvCesA2_BART1_0p40943007
HvCesA8_BART1_0p36668020
HvCesA8_BART1_0p36668002
HvCesA8_BART1_0p36668003
HvCslF10_BART1_0p10215002
HvCslF12_BART1_0p10200001
HvCslF10_BART1_0p10215004
HvCslF10_BART1_0p10215001
HvCslF10_BART1_0p10215003
HvCslF10_BART1_0p10215005
HvCslJ_BART1_0p18352001
HvCslH1_BART1_0p12403001
HvCslH1_BART1_0p12403003
HvCslF9_BART1_0p01715001
HvCslF3_BART1_0p10207001
HvCslF7_BART1_0p33872001
HvCslF9_BART1_0p01715002
HvCesA7_BART1_0p22208014
HvCesA7_BART1_0p22208008
HvCesA7_BART1_0p22208015
HvCesA2_BART1_0p40943009
HvCslF11_BART1_0p57215001
−1
−0.75
−0.5
−0.25
0
0.25
0.5
0.75
1

Figure S9. Correlation of expression (TPM values) of wider set of genes in all 807 samples (eoRNA datasets; Milne *et al*., 2021) ordered based on hierarchical clustering. Each transcript is represented by its corresponding BART1_ ID.

References

Criscuolo, A., and Gribaldo, S. (2010). BMGE (Block Mapping and Gathering with Entropy): A new software for selection of phylogenetic informative regions from multiple sequence alignments. *BMC Evol. Biol.* 10. doi:10.1186/1471-2148-10-210.

Kumar, S., Stecher, G., Li, M., Knyaz, C., and Tamura, K. (2018). MEGA X: Molecular evolutionary genetics analysis across computing platforms. *Mol. Biol. Evol.* 35, 1547–1549. doi:10.1093/molbev/msy096.

Pucker, B. (2022). Automatic identification and annotation of MYB gene family members in plants. *BMC Genomics* 23, 1–11. doi:10.1186/s12864-022-08452-5.
